# Optical Detection of Distal Lung Enzyme Activity in Human Inflammatory Lung Disease

**DOI:** 10.34133/2021/9834163

**Published:** 2021-04-07

**Authors:** Alicia Megia-Fernandez, Adam Marshall, Ahsan R. Akram, Bethany Mills, Sunay V. Chankeshwara, Emma Scholefield, Amy Miele, Bruce C. McGorum, Chesney Michaels, Nathan Knighton, Tom Vercauteren, Francois Lacombe, Veronique Dentan, Annya M. Bruce, Joanne Mair, Robert Hitchcock, Nik Hirani, Chris Haslett, Mark Bradley, Kevin Dhaliwal

**Affiliations:** ^1^ EaStCHEM, The University of Edinburgh School of Chemistry, Joseph Black Building, West Mains Road, Edinburgh, UK, EH9 3FJ; ^2^ Translational Healthcare Technologies Group, Centre for Inflammation Research, Queen’s Medical Research Institute, University of Edinburgh, 47 Little France Crescent, Edinburgh BioQuarter, Edinburgh, UK, EH16 4TJ; ^3^ The Roslin Institute and Royal (Dick) School of Veterinary Studies, University of Edinburgh, Easter Bush, Midlothian, UK, EH25 9RG; ^4^ Department of Biomedical Engineering, University of Utah, 36 S Wasatch Dr, Salt Lake City, UT 84112, USA; ^5^ School of Biomedical Engineering & Imaging Sciences, King’s College London, London, UK, SE1 7EH; ^6^ Mauna Kea Technologies, 9, Rue d’Enghien, Paris, France75010

## Abstract

*Objective and Impact Statement.* There is a need to develop platforms delineating inflammatory biology of the distal human lung. We describe a platform technology approach to detect *in situ* enzyme activity and observe drug inhibition in the distal human lung using a combination of matrix metalloproteinase (MMP) optical reporters, fibered confocal fluorescence microscopy (FCFM), and a bespoke delivery device. *Introduction*. The development of new therapeutic agents is hindered by the lack of *in vivo in situ* experimental methodologies that can rapidly evaluate the biological activity or drug-target engagement in patients. *Methods*. We optimised a novel highly quenched optical molecular reporter of enzyme activity (FIB One) and developed a translational pathway for in-human assessment. *Results*. We demonstrate the specificity for matrix metalloproteases (MMPs) 2, 9, and 13 and probe dequenching within physiological levels of MMPs and feasibility of imaging within whole lung models in preclinical settings. Subsequently, in a first-in-human exploratory experimental medicine study of patients with fibroproliferative lung disease, we demonstrate, through FCFM, the MMP activity in the alveolar space measured through FIB One fluorescence increase (with pharmacological inhibition). *Conclusion*. This translational *in situ* approach enables a new methodology to demonstrate active drug target effects of the distal lung and consequently may inform therapeutic drug development pathways.

## 1. Introduction

Respiratory diseases are one of the leading causes of morbidity and mortality worldwide [1]. Despite the significant burden of disease, there is a paucity of new pharmacological moieties reaching clinical phases of development and even fewer reaching clinical approval with spiraling costs of drug development [2]. Furthermore, the recent COVID-19 global pandemic has highlighted the need for rapid approaches to assess drugs that cause respiratory morbidity and mortality [3].

To date, the evaluation of early-phase clinical trials in human pulmonary disease has relied upon measurements, which include pulmonary function measurements, six-minute walk tests, and imaging such as computerized tomography (CT) [4]. These investigations can inform on functional and structural abnormalities but are delayed surrogates of active disease and offer little insight into dynamic molecular processes where therapeutics may be beneficial. Optical imaging modalities provide the potential for rapid readouts of disease activity at the site of pathogenesis, such as the distal lung [5]. Therefore, we developed a novel optical imaging approach using a combination of an existing miniaturized fibred confocal fluorescent microscopy (FCFM) system, a bronchoscope compatible delivery device, a new molecular probe, and a drug inhibitor to demonstrate real-time drug-target engagement in the distal lung of patients.

The matrix metalloproteinase (MMP) pathway was selected for detection and inhibition within a small patient population (eight patients with predicted aberrant lung fibrogenesis) to demonstrate the clinical tractability of our approach. MMPs are mediators of injury and repair, performing pivotal roles in the turnover of the extracellular matrix (ECM), with dysregulation leading to an aberrant response in inflammation and tissue repair, which ultimately leads to an impairment of organ function [6–9]. MMP dysregulation has been associated with many acute and chronic inflammatory respiratory diseases such as acute respiratory distress syndrome (ARDS) [10], idiopathic pulmonary fibrosis (IPF) [11], chronic obstructive pulmonary disease (COPD) [12], and lung cancer [13], with upregulation of the gelatinases (MMP-2 and MMP-9) often found in IPF [9, 14] and associated with a more aggressive disease phenotype [15]. The collagenase MMP-13 has also been shown to be abundant in the IPF tissue, [16] and as such, there has been considerable interest in the assessment of pulmonary MMP activity as a biomarker of fibrotic lung disease [17, 18] and in the therapeutic targeting of MMP activity in acute and chronic lung disease [19, 20] and the malignant matrix [21, 22].

The aim of this exploratory translational study was to develop and assess a novel optical reporter that was able to image MMP activity (measured though fluorescence increase of FIB One) within the alveolar space and demonstrate drug-target engagement using a microdosing approach of a codelivered MMP inhibitor (moieties delivered being less than 100 *μ*g of imaging agent or drug). This methodology and the demonstration of real-time drug-target engagement *in situ* allowed the bedside assessment of pharmacological action in human disease using optical imaging.

## 2. Results

### 2.1. A Tribranched Scaffold Is Efficiently Cleaved by MMPs with Amplification of Fluorescence Signal within Minutes

We recently described a MMP peptide substrate [23, 24] that was selectively and specifically cleaved by secreted MMPs 2, 9, and 13 resulting in fluorescent signal amplification and designed to be resistant to plasmin and other bystander enzymes. However, although highly selective, this linear optimised probe demonstrated low signal-to-noise following cleavage.

**Figure 1:**
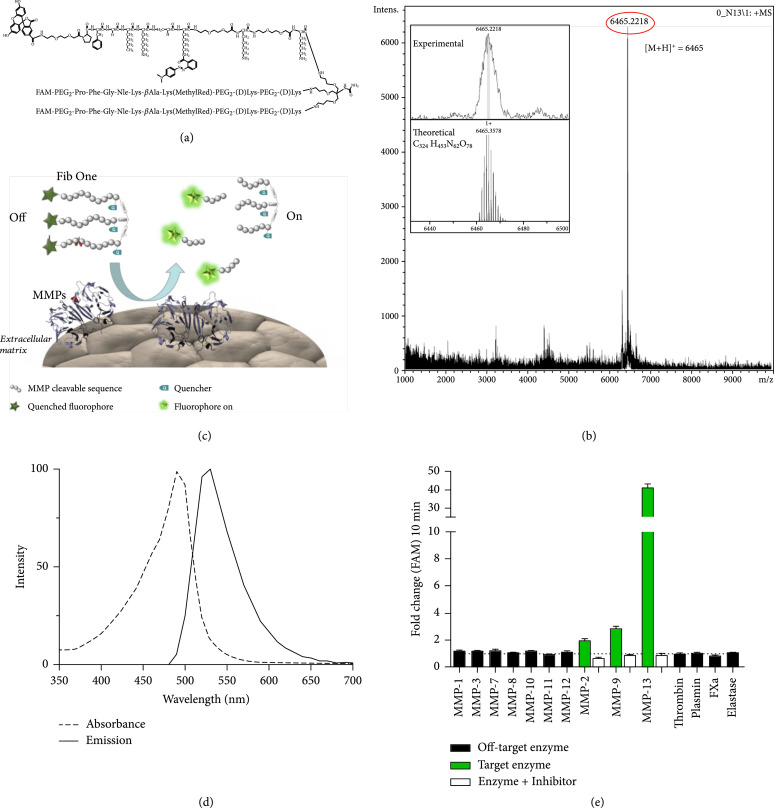
Structure, characterisation, mode of action, and in vitro validation of FIB One. (a) Chemical structure of FIB One. (b) FIB One characterisation by MALDI-ToF MS analysis: m/z 6465 [M+H]^+^). (c) The FIB One structure showing how fluorescence switches from ‘off’ to ‘on’ following cleavage by the target MMP’s. (d) Absorbance and emission spectra of FIB One. (e) Fold-change in fluorescent signal of FIB One (1 *μ*M) following 10 min incubation with target (green) and off-target (black) enzymes, compared to enzyme-free control. Activation of FIB One by target enzymes was prevented by the addition of the MMP inhibitor marimastat (20 *μ*M, white). Data shows mean and s.e.m. n=3 performed in duplicate, MMPs 30 nM, thrombin 5 U/mL, plasmin 30 nM, factor Xa 500 nM, and excitation/emission 485/528 nm.

To maximise the fluorescence amplification, we modified the linear probe by using a multiple Förster resonance energy transfer (FRET) system with insertion of the substrate peptide sequence into a tribranched scaffold. This tribranched structure takes advantage of both the self-quenching effect observed in multifluorophore constructs [25–27] as well as the quenching effect of three quenchers [28]. The molecular probe generated (called FIB One (Figure 1)) consisted of a tribranched peptide (Pro-Phe-Gly-Nle-Lys-*β*Ala)_3_, containing three fluorescein units and three methyl red groups (Figure 1). FIB One was prepared using Fmoc-based solid-phase peptide synthesis (Scheme S1 and ESI), and after purification, was fully characterized by reverse-phase high-pressure liquid chromatography (RP-HPLC) and matrix-assisted laser desorption ionising time-of-flight mass spectrometry (MALDI-ToF MS) (Figure 1(b) and ESI).

Selective hydrolysis of the peptidic sequence by the active enzyme results in an increase in fluorescence due to the release of the fragments containing fluorescein (Figure 1(c)), with excitation and emission maxima at 490/530 nm, respectively (Figure 1(d)). FIB One demonstrated rapid and specific fluorescent amplification when exposed to recombinant enzymes (MMPs 2, 9, and 13), which was blocked by the pan-MMP inhibitor marimastat (Figure 1(e) and Figure S1), with MALDI-ToF MS showing confirmation of selective cleavage (Figure S1). In addition, comparison of FIB One with its analogous monomeric linear structure confirmed improved signal amplification obtained with the multimeric approach (Figure S2).

### 2.2. FIB One Is Cleaved by MMPs in Ex Vivo Primary Human Models and in an Ex Vivo Lung Model

**Figure 2:**
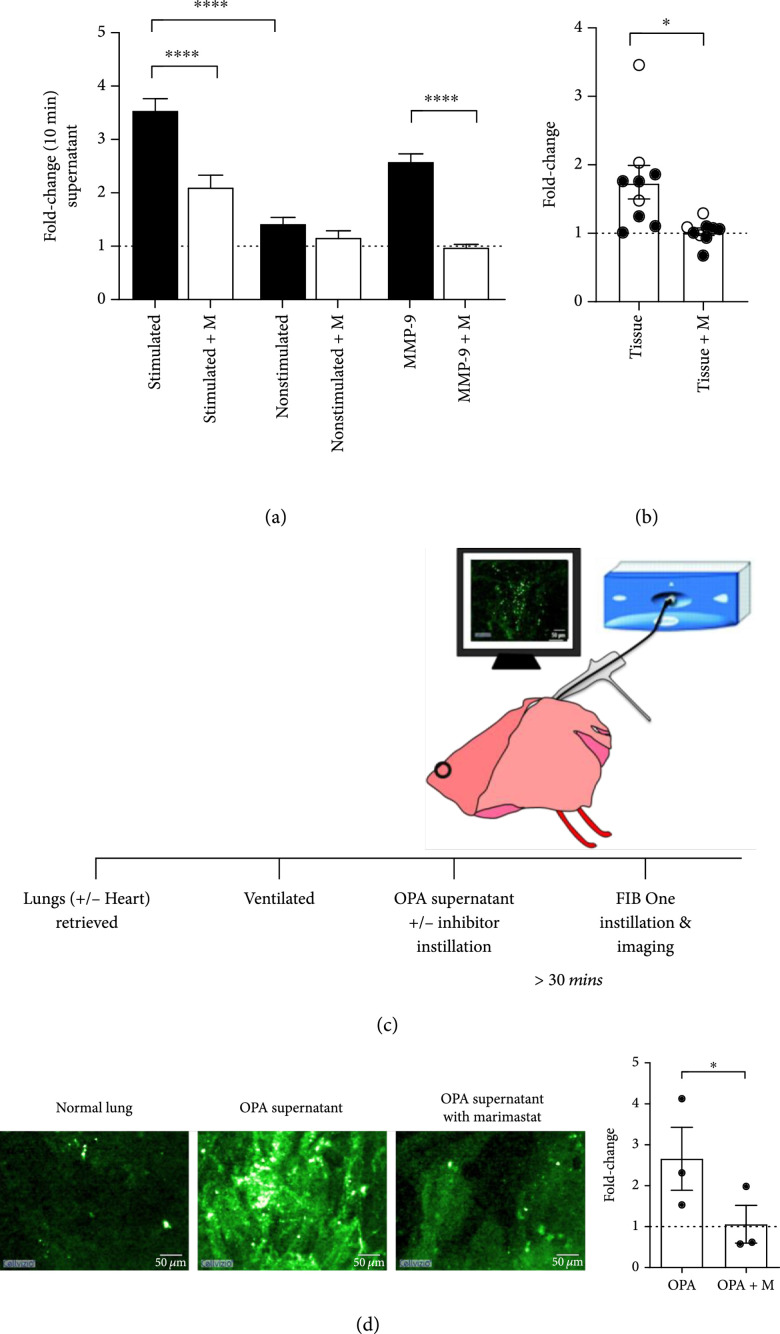
Validation of FIB One in physiologically relevant biological models. (a) Fold-change in FIB One (1 *μ*M) signal following 10 min incubation with supernatant from stimulated or nonstimulated neutrophils *in vitro* with and without a pan-MMP inhibitor, marimastat (M). Recombinant human MMP-9 (30 nM) shown as reference. Bars show mean (+/-SEM), n≥4 for each condition, analysis by one-way ANOVA ${}^{****}P<0.0001$. MALDI-ToF MS demonstrating site-specific probe cleavage in ESI Figure S3a. (b) Quantification from e*x vivo* fibre-based imaging of aged human lung tissue (open circles of pulmonary fibrosis patients and black circles of aged nonfibrotic lung tissue) with an FCFM imaging system (488 nm excitation). The fluorescence from samples with FIB One (1 *μ*M) or FIB One plus marimastat (100 *μ*M) was imaged (10 min) and quantified. Graph shows fold-change compared to baseline autofluorescence of the same sample. Data show mean (+/-SEM), n=9, ${}^{*}P=0.0215$, student’s paired t-test. (c) Experimental set-up of size-relevant *ex vivo* ovine pulmonary adenocarcinoma (OPA) sheep lung model. (d) Representative images of *ex vivo* ovine lung and OPA segments following administration of FIB One with and without inhibitor. Scale bar represents 50 *μ*m. Graph demonstrates quantification of all experiments relative to intrinsic autofluorescence. n=3, bars show mean (+/-SEM), analysis by paired t-test for each normalised pre-post-FIB One segment, ${}^{*}P=0.0476$.

To determine the clinical tractability of this approach and for the compound to be cleaved at physiologically relevant/disease levels of MMPs, we evaluated the propensity for FIB One to be specifically cleaved by MMPs 2, 9, and 13 in primary human lung tissue and also by stimulated human neutrophils. FIB One was cleaved in both these scenarios with cleavage inhibition observed following coincubation with marimastat (Figures 2(a) and 2(b)). MALDI-ToF MS confirmed that the cleavage in these models was MMP specific (Figure S3).

The translational utility required evidence that the MMP activity could be rapidly detected in the distal lung in real time (within seconds to minutes) using an endoscopic approach. Therefore, we utilised an *ex vivo* ovine lung model (Figure 2(d)). Here, to demonstrate MMP upregulation, segments instilled with supernatant from spontaneously occurring ovine pulmonary adenocarcinoma (OPA) enabled evaluation of FIB One (Figure 2(c)). FCFM-based imaging of segments with high MMP activity (Figure S3) confirmed a diffuse pattern of increased fluorescence across the field of view, which was a characteristic of cleaved FIB One (Figure 2(d)). In contrast, vehicle control segments and marimastat (pan-MMP inhibitor)-dosed segments did not show an increase in FIB One signal (Figure 2(d)). Together, these data supported that pulmonary delivery of FIB One could detect MMP activity *in situ* when coupled with FCFM within a size relevant model within physiological concentrations.

### 2.3. GMP-Manufactured FIB One Demonstrated Stability, Lack of Toxicity, and Favourable Alveolar Imaging in Fibrotic Lung Disease in Human Patients

The translational pathway required toxicological and functional evaluation of FIB One. FIB One was synthesised in accordance with active pharmaceutical ingredient (API) development to good manufacturing practice (GMP) principles as stated in Eudralex [29]. It was prepared on a 100 mg scale and demonstrated no degradation as an aqueous formulation over 12 months (Figure S4 and Table S8). FIB One demonstrated no overt biological toxicity, as evidenced by absence of erythrocyte hemolysis, and no preclinical *in vivo* toxicity, supported by a rodent intratracheal instillation model of high FIB One concentrations (500 *μ*g/mL, compared to 20 *μ*g/mL for intended human pulmonary dosing), which resulted in no pulmonary inflammatory cell recruitment, pulmonary toxicity, or systemic toxicity at either early or late time points (Table S7).

**Figure 3:**
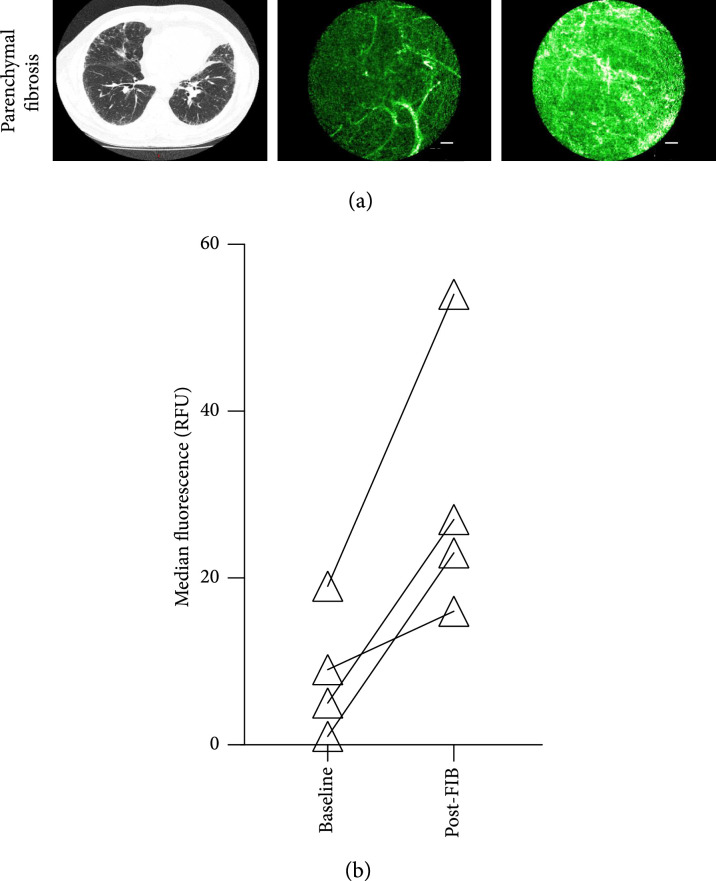
Imaging of parenchymal fibrosis following delivery of FIB One endobronchially. (a) Representative CT images (axial image from a patient with rheumatoid arthritis associated interstitial lung disease) with baseline and post-FIB One images acquired by confocal endomicroscopy imaging. (b) Quantification of *in vivo* human lung fluorescence measured using confocal endomicroscopy before and after bronchial delivery of the FIB One (n=4 for each group, P>0.05). Statistical analysis using a Mann–Whitney test. Scale bar represents 50 *μ*m.

Using a conventional catheter in the working channel of a bronchoscope, we delivered FIB One endobronchially as a bolus to the lungs of eight patients with fibroproliferative lung disease in which we expected high MMP level (endobronchially visible tumours and diffuse pulmonary fibrosis). Alveolar FCFM imaging following endobronchial delivery of the probe resulted in increased fluorescent signal in all patients with parenchymal fibrosis (Figure 3). For cancer imaging, *ex vivo* data suggested that MMP activity utilising FIB One could be imaged but *in vivo* imaging of bronchial tumours was complicated by FIB One washing off the bronchial mucosa rapidly (Figure S5). There were no significant adverse events in the eight patients (Supplementary tables S1-S6).

### 2.4. A Triple Lumen Bronchoscopy Catheter (TLBC) Enabled Direct Imaging of MMP Activity in the Alveolar Space in Patients which Was Inhibited by Codelivery of a MMP Inhibitor

Although we detected MMP activity in patients with active fibrotic lung disease, our approach was hampered by requiring the delivery of a proximal bolus of FIB One down a conventional catheter in a step immediately preceding insertion of the imaging fibre. To enable concurrent FIB One delivery and imaging at the exact same location, we developed and deployed a triple lumen bronchoscopy catheter (TLBC) [30] which allows delivery of compounds and simultaneous FCFM imaging in the field of view.

**Figure 4:**
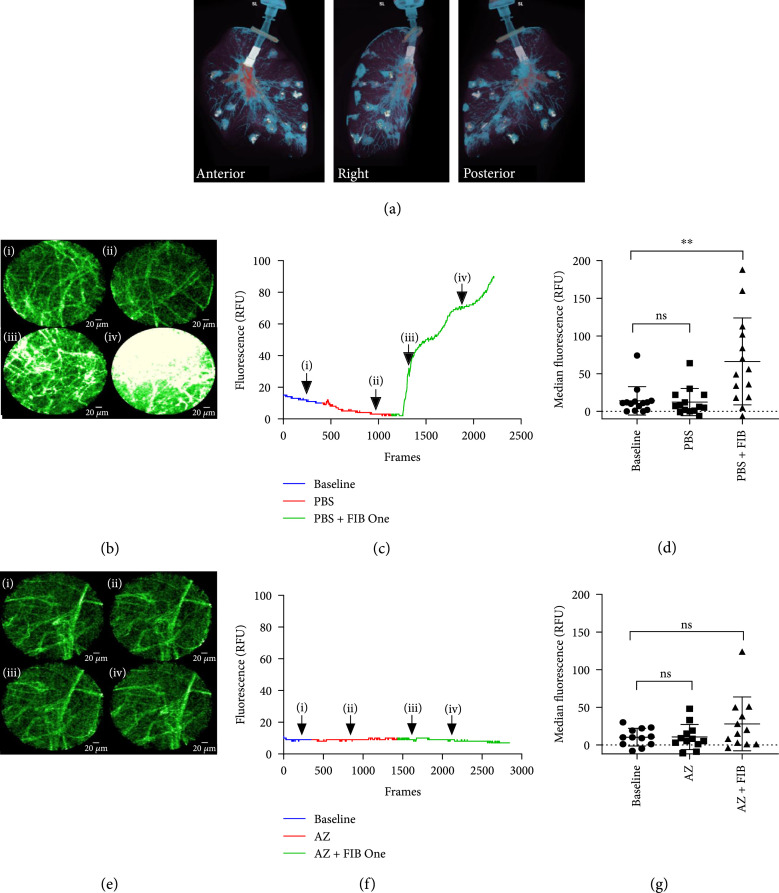
*In vivo* evidence of MMP activity with pharmacological inhibition in patients with pulmonary fibrosis. (a) CT images of an inflated *ex vivo* human right lung with 500 *μ*L aliquots of iodinated contrast delivered transbronchially. (b) Representative *in vivo* images from a patient with IPF: (i) alveolar space before administering any agents, (ii) after administering PBS, and (iii, iv) after the administration of FIB One. Dynamic range is identical for all images. Scale bar represents 20 *μ*m. (c) Mean fluorescence of field of view over time during administration of PBS and FIB One. Corresponding time points to the images in (b) are indicated with arrows. (d) Median fluorescence of each video from 14 pulmonary segments (^ns^P=0.36, ${}^{**}P=0.002$). Error bars represent mean+/−s.d and statistical values from Wilcoxon matched pairs. (e) Representative images from a different pulmonary segment in the same patient as in (b): (i) alveolar space before administering any agents, (ii) after administering MMP inhibitor (AZD1236), and (iii, iv) after the administration of FIB One. Dynamic range is identical for all images. (f) Mean fluorescence of field of view over time during administration of AZD1236 and FIB One. Corresponding time points to the images in (e) are indicated with arrows. (g) Median fluorescence of each video from 12 pulmonary segments dosed with AZD1236 (P=0.51) or AZD1236 plus FIB One (P=0.05). Error bars represent mean+/−s.d and statistical values from Wilcoxon matched pairs. RFU: relative fluorescence unit; PBS: phosphate-buffered saline; AZ: AZD1236 MMP inhibitor.

To assess the distribution of agents delivered to the lung parenchyma, we undertook preclinical testing of the TLBC in an *ex vivo* ventilated human lung model. 500 *μ*L aliquots of iodinated contrast agent were delivered into the alveolar space and distribution assessed by CT scan, demonstrating a dispersal volume of 0.5 cm^3^ (Figure 4(a)). The TLBC offered the capability to codeliver microdoses of a drug inhibitor. Thus, we reformulated an orally bioavailable small molecule inhibitor of MMPs (AZD1236) which had been previously evaluated in Phase-II trials into a GMP aqueous drug product and evaluated its toxicological and biological activity (Figure S6 Table S9).

Using this approach, we obtained real-time imaging sequences in nine patients with inflammatory fibroproliferative disease before and after FIB One delivery, with and without localised predelivery of AZD1236 (Figure 4, Table S5 and Figure S6), demonstrating *in situ in vivo* drug-target engagement within the alveolar space.

## 3. Discussion

We report the first in-human pulmonary optical molecular imaging of enzyme activity, through fluorescence increase of an MMP reporter, and dynamic elucidation of drug-target engagement in the distal alveolar regions of the human lung.

The FIB One probe was specific to MMPs 2, 9, and 13 which degrade gelatin, type IV collagen, and elastin within the ECM, which are known to be abundant in the context of acute and chronic inflammation [31] where they are secreted by infiltrating inflammatory cells as well as bronchial and alveolar epithelial cells and fibroblasts [32]. To ensure future validity of our work, there would need to be inclusion of a noninflammatory control group to determine the detection of upregulated vs. homeostatic MMP activity. However, if confirmed, MMPs represent an attractive target as they serve as a marker in several human inflammatory diseases including IPF, COPD, ARDS, and lung cancer [10–14]. With the advent of increasingly available multiplexing approaches for microscopic interrogation, our approach of using fluorescent reporters with FCFM and drug inhibition does represent a wider applicability and is readily extendable to multiple target pathways in several organ systems.

Current approaches to identifying enzyme activity in the lung has been challenging and has typically been performed *ex vivo* using zymography on patient tissue or lavage samples [33]. However, this technique has several limitations, including the requirement for an invasive biopsy and tissue retrieval and the utilisation of lavage fluid, which is prone to sampling inconsistencies. Furthermore, zymography assessment may not reflect free enzyme activity in the native environment, which in the case of MMPs, is dependent on inhibitory antagonists such as tissue inhibitors of MMPs (TIMPs).

Molecular imaging of enzyme activity and pharmacological perturbation *in vivo* has focused on either nuclear imaging of radiolabelled probes or optical imaging with fluorogenic probes. These studies have used animal models to image MMP activity *in vivo* in cancer [34–39], stroke [40], and joint disease [41–43]; however, they have all relied on systemic intravenous delivery of the probes and whole-body optical imaging, which is to date, technically not possible in humans with sufficient resolution or sensitivity. Thus, we adopted an alternate approach to measure enzyme activity in the distal lung human lung, made possible by using FCFM to allow us to access alveolar regions and deliver microdoses (<100 *μ*g) of FIB One directly to the region of interest without concerns of toxicity. It was of importance that the area of tissue imaged coincided with the delivery of the imaging reagent (FIB One). Whilst our initial endobronchial delivery yielded encouraging results, we could not eliminate the possibility that FCFM fibre placement would miss a predosed alveolar region. Our preclinical data supports the use of this approach in lung cancer, although there were technical difficulties with imaging endobronchial tumours with rapid dissipation of probe. These may be mitigated in the future with further adaptation to the technical approach or modifications to the probe to allow cancer cell labelling [24].

MMP inhibition has been identified as a target for drug interventions in inflammatory diseases [44, 45]. MMP inhibitors used in trials as cancer therapies were disappointing due to lack of efficacy and significant adverse effects [46]. Studies have also been limited by a lack of quantifiable biomarkers to reflect on their *in vivo* and functional activity [20]. In this study, we reformulated and toxicologically qualified AZD1236, an orally available MMP-9/12 inhibitor which also demonstrated an ability to inhibit MMPs 2 and 13. It had previously been used in a phase 2A safety and tolerability study in 74 emphysema patients randomised to placebo or AZD1236 75 mg twice daily for 6 weeks [19]. The study demonstrated no effect on the secondary lung physiology endpoints, and the investigators were unable to show that AZD1236 actually inhibited its intended targets *in situ*. Indeed, a placebo-controlled, AZD1236 biomarker study of 55 COPD patients again failed to demonstrate MMP-9 or MMP-12 inhibition in induced sputum [20], whereas we have demonstrated drug-target engagement of AZD1236 in humans.

The pathway of drug validation utilises cell-based assays and animal models of disease followed by phased studies in humans. At each stage of development, costs increase and there is a high attrition rate often leading to the failure or deescalation of drug candidates. Although there are many reasons for this, one major hurdle is demonstrating that enzymatic activity is present in the patients being studied. Therefore, there is a rationale for performing translational proof of concept (experimental medicine) studies directly and rapidly in humans, using small numbers of patients. Consequently, here, we have described a tractable strategy for rapidly assessing distal alveolar biology using a sensitive and specific optical probe and direct visualisation of MMP pathway activity/inhibition *in situ* in real time with FCFM. In the future, this could be assessed in a broader group of patients with inflammatory and noninflammatory lung disease correlated with the MMP activity and may present the ability to offer a mechanism-based method of stratifying patients for drug studies and to interrogate biological pathways *in situ in vivo* with molecular microscopy.

## 4. Materials and Methods

### 4.1. Ethics Statement

All experiments using human samples *in vitro* were performed following approval of the appropriate regional ethics committee (REC) and with informed consent of the patients. Blood sample collection and use was approved by Lothian REC No: 08/S1103/38 and 15/HV/013. Tissue samples were obtained following approval by NHS Lothian REC and facilitated by NHS Lothian SAHSC Bioresource REC No: 13/ES/0126 and NHS Lothian REC No: 09/S1101/52. The *ex vivo* human lungs were deemed unsuitable for transplant and authorised for research as part of the Enlighten study (London-Central Research Ethics Committee, REC Reference: 16/LO/1883). The in-human assessments were undertaken with informed consent of the participants and approved by the South East Scotland Regional Ethics Committee 02, REC Number: 15/SS/0235. The trial was registered with ClinicalTrials.gov (Identifier: NCT02604862).

### 4.2. In Vitro Enzymatic Validation of FIB One

To determine the enzymatic specificity of FIB One, the probe was incubated at a concentration of 1 *μ*M (unless otherwise stated) with the recombinant MMPs (Enzo Life Sciences) (active domains of MMP-1, MMP-2, MMP-3, MMP-7, MMP-8, MMP-9, MMP-10, MMP-11, MMP-12, and MMP-13, all at 30 nM), thrombin (Sigma Aldrich) (5 U/mL), factor Xa (Sigma Aldrich) (500 nM), plasmin (Sigma Aldrich) (30 nM), and human neutrophil elastase (30 nM). Where appropriate, enzymes were incubated with specific inhibitors for 30 min at 37°C prior to addition of FIB One. The pan-MMP inhibitor marimastat (Tocris Biosciences) was used at 20 *μ*M, and the MMP inhibitor AZD1236 was used at 0-14 *μ*M. Enzyme free reactions served as a control. Enzymatic reactions were performed in MMP buffer (10 mM CaCl_2_, 6.1 g Tris-HCl, 8.6 g NaCl per litre, pH 7.5) in a final volume of 20 *μ*L. Reactions were performed in duplicate in 384-well plates (Life Technologies) with optically clear plate seals (Thermo Scientific) and repeated thrice (independently). Fluorescence signal was measured for up to 60 min, ex/em 485/528 nm using microplate reader (BioTek Synergy H1 multimode reader). Data were normalised to buffer alone and are presented as fold-change in signal (relative fluorescent units, RFU) compared to enzyme-free controls. Data were plotted using Prism 7 (GraphPad Software Inc., La Jolla, CA, USA).

### 4.3. Neutrophil Extraction and Evaluation of FIB One

Neutrophils were isolated from the blood of healthy human volunteers as previously described [47]. The number of retrieved neutrophils was determined with NucleoCounter NC-1000 (Chemo Metec). Neutrophils were resuspended at a concentration of $20\times 10^{6}$ mL^-1^ in 0.9% NaCl with 0.9 mM CaCl_2_. Cells were incubated for 30 min at 37°C. Cells to be stimulated were subsequently activated with 5 *μ*M calcium ionophore A23187 (Tocris Bioscience) for 30 min at 37°C. Neutrophils were harvested by centrifugation at 400 x g for 5 min. Supernatants were removed and stored. Plate reader assays were performed as described above, with the MMP buffer replaced with neutrophil supernatant. All experiments were carried out in duplicate using three independent donors (one donor per independent repeat). Data were normalised by background subtraction of intrinsic fluorescence.

### 4.4. Ex Vivo Human Lung Tissue Processing and Evaluation of FIB One

Human lung tissue samples (lung tumour and noncancerous adjacent tissue) were obtained following surgical resection and stored at -80° C until use. Processing for multiwell plate reader assay or zymography analysis was conducted as follows: 1 mm×4 mm sections were homogenised in 400 *μ*L MMP buffer in Precellys 2.8 mm ceramic bead tubes (VWR) using a Pecellys 24 homogeniser. Homogenised tissue was adjusted to 4 mg mL^-1^ protein (determined by a Pierce BCA Total Protein Assay Kit (Thermo Fisher Scientific), following the manufacturer’s instructions). Multiwell plate reader assays were performed as previously described, replacing the MMP buffer and MMP enzymes for the homogenised and protein-adjusted lung tissue.

For zymography, the samples were mixed 1 : 1 with 2x SDS sample buffer and 20 *μ*L was loaded onto precast Novex® 10% Zymogram (Gelatin) Protein Gels (Thermo Fisher Scientific). The gels were placed in an electrophoresis chamber with pre-chilled Novex diluted running buffer and electrophoresed at 150 V for approximately 90 min at 4°C. Gels were removed and incubated with Novex renaturing buffer for 90 min at 4°C. They were washed in distilled water and incubated with Novex developing buffer for 30 min at room temperature prior to overnight incubation at 37°C. Control gels had marimastat (50 *μ*M) added to the developing buffer. Gels were then rinsed with distilled water prior to staining with a colloidal blue staining kit and imaging using a transilluminator (UVItec BXT-20 M, UVItec Ltd, Cambridge, UK).

For *ex vivo* imaging, human lung tissue was sliced into 1 mm×4 mm sections and placed in the wells of a 96-well plate with 100 *μ*L of MMP buffer and 30 nM MMP-13. Where appropriate, AZD1236 (0-14 *μ*M as indicated in the text) was added to the tissue and incubated at 37°C for 30 min prior to the addition FIB One (5 *μ*M). The tissue was imaged over 10 min, 12 frames sec^-1^ using a preclinical confocal laser scanning endomicroscopy device (Cellvizio®, Mauna Kea Technologies, excitation 488 nm/detection bandwidth 505 to 700 nm) with a compatible imaging fibre (Alveoflex™, Mauna Kea Technologies). At each time point 60 s, images of captured with same laser power. The fluorescence for each sample was calculated by determining the average fluorescence per frame for each time point. All images were brightness and contrast enhanced with the same parameters. Five independent patient specimens were collected and analysed. RFUs collected from off-target frames (showing motion artefact or absence of any fluorescence) were omitted during analysis.

### 4.5. Ex Vivo Lung Experimental Procedure

Ovine pulmonary adenocarcinoma (OPA) tissue was homogenised using a drill homogeniser, followed by lysis in cell lysis buffer. The supernatant was collected following centrifugation at 12,000 rpm for 20 min. The samples were adjusted to 1 mg mL^-1^ total protein (determined by a Pierce BCA Total Protein Assay Kit (Thermo Fisher Scientific)). Zymography (as described above) was performed to confirm MMP activity across multiple OPA samples. Recombinant MMP-9 (2 nM) and recombinant pro-MMP-9 (0.5 nM) were included in the assay as positive controls.

OPA supernatant (1 mL) and marimastat (100 *μ*M) or 1 mL 0.9% NaCl (control) was instilled into anatomically distinct segments of *ex vivo* ventilated non-OPA ovine lungs. Following >30 min, the segments were bronchoscopically identified and microdosed with 5 *μ*M FIB One in 1 mL. FCFM imaging was undertaken pre- and postinstillation (Cellvizio®, Mauna Kea Technologies). This was performed by passing a FCFM fibre into the disparate bronchopulmonary segments (up to 5 passes per bronchopulmonary segment to capture a regional representation of fluorescence), recording images at 12 frames per second and imaging for up to 5 minutes. Videos were analysed as described above. Each experimental set was normalised to the control segment (segment with 1 mL 0.9% NaCl instilled) which represents intrinsic autofluorescence.

### 4.6. Human Ex Vivo Experimental Procedure

The development of the TLBC has been described previously [30]. This device was compatible with a slimmer (diameter 1 mm) imaging fibre approved for clinical investigation (Miniaturized AlveoFlex™ Confocal Miniprobe, Cellvizio®, Mauna Kea Technologies) and had two hollow lumens to allow FIB One and/or AZD1236 drug delivery directly to tissue within the field of view during image acquisition. The TLBC device was validated preclinically within ventilated *ex vivo* human lungs to assess both fluid delivery capability and to determine which segments of the lung the device was able to reach. Iodinated contrast (Omnipaque 300, GE healthcare) was diluted with PBS in a ratio of 1 : 5 and delivered transbronchially in 500 *μ*L doses through the device lumens to the distal lung, repeated for multiple pulmonary segments. Following delivery, the lung was inflated, the trachea clamped, and computerized tomography (Biograph mCT, Siemens) images were taken to visualise the extent of alveolar contrast spread. Processing was performed with Horos DICOM viewer (HOROS).

### 4.7. Clinical Study Patient Selection

All participants were scheduled for routine, clinical diagnostic flexible bronchoscopy in our institution with a known or suspected diagnosis of fibrosis or lung cancer. All eight patients had computerized tomography performed prior to bronchoscopy and regions of active disease identified for targeted investigation.

### 4.8. Bronchoscopy and FCFM Procedure

Bronchoscopy was undertaken in an outpatient suite using local anaesthetic and conscious sedation or in theatre under general anaesthetic. FCFM was performed using a clinically approved confocal laser scanning endomicroscopy device (Cellvizio®, Mauna Kea Technologies) with a compatible imaging fibre (Alveoflex™ or Miniaturized Alveoflex™, Mauna Kea Technologies).

In initial exploratory testing, 8 patients with either endobronchial tumour or parenchymal fibrosis underwent FIB One microdosing (<100 *μ*g, <5 mL). FIB One was administered using a delivery catheter (ERBE APC catheter or TLBC) passed out of the working channel of the wedged bronchoscope into the bronchus allowing distal dissipation of the compound for alveolar imaging or applied directly onto visible tumour. FCFM imaging was performed pre- and postinstillation of FIB One, with the fibre tip positioned at the bronchial mucosa or passed into the alveolar region following transbronchial puncture, as appropriate for the clinical indication. Images were recorded for at least 30 s at 12 frames/second.

### 4.9. In Vivo Assessment of FIB One and AZD1236 with TLBC

In subsequent testing the Miniaturized Alveoflex™-TLBC combination replaced the Alveoflex™ fibre. The entire apparatus was passed transbronchially to allow direct alveolar imaging before localised delivery of agents. Once transbronchial pass was accomplished, baseline lung imaging was recorded for at least 30 sec. A 500 *μ*L microdose of either AZD1236 (14 *μ*g/mL) or sterile PBS (control) was administered under direct observation, and further imaging was obtained for >60 sec. 500 *μ*L of FIB One (20 *μ*g/mL) (delivered only after at least 60s to allow MMP inhibition) was then dosed to the same area with a further 90-120 sec of imaging captured. If considerable movement due to breathing or coughing was encountered, imaging was halted and a new segment identified. Up to 6 segments of interest were investigated per patient determined by patient tolerance.

### 4.10. Image Analysis

All video sequences were assessed by two clinicians with clinical experience of pulmonary endomicroscopy. Frames with excessive movement or lack of recognizable alveolar structure were removed from analysis. Videos were assessed to ensure stability throughout the imaging procedure. To ensure the region imaged was the same as region dosed, imaging from a segment was excluded if there was obvious alveolar movement witnessed during image capture. All video files were analysed using the Cellvizio® Viewer image processing software from Mauna Kea Technologies, Paris. A region of interest was drawn around the fibre field of view and processed to determine the mean RFU for each frame. The videos were categorized as either baseline, post-PBS, post-AZD1236, post-FIB One, and PBS or post-FIB One and AZD1236 for each patient. The median RFU was calculated for each category and used as a comparative value between groups.

### 4.11. Statistical Analysis

Statistical analyses were performed using Prism 7 (GraphPad Software Inc., La Jolla, CA, USA). Where appropriate, analyses were performed using the student’s t-test or one-way ANOVA. Unless otherwise stated error bars show standard error of the mean (s.e.m).

## Data Availability

The authors confirm that the data supporting the findings of this study are available within the article and its supplementary materials.

## Supplementary Materials

Supplementary Materials Supplementary Information containing supplementary Figures S1-6: chemistry materials and methods, patient data, toxicology studies, and stability studies.
