# Erratum to “Optical Detection of Distal Lung Enzyme Activity in Human Inflammatory Lung Disease”

**DOI:** 10.34133/bmef.0029

**Published:** 2023-09-20

**Authors:** Alicia Megia-Fernandez, Adam Marshall, Ahsan R. Akram, Bethany Mills, Sunay V. Chankeshwara, Emma Scholefield, Amy Miele, Bruce C. McGorum, Chesney Michaels, Nathan Knighton, Tom Vercauteren, Francois Lacombe, Veronique Dentan, Annya M. Bruce, Joanne Mair, Robert Hitchcock, Nik Hirani, Chris Haslett, Mark Bradley, Kevin Dhaliwal

**Affiliations:** ^1^ EaStCHEM, The University of Edinburgh School of Chemistry, Joseph Black Building, West Mains Road, Edinburgh EH9 3FJ, UK.; ^2^ Translational Healthcare Technologies Group, Centre for Inflammation Research, Queen’s Medical Research Institute, University of Edinburgh, 47 Little France Crescent, Edinburgh BioQuarter, Edinburgh EH16 4TJ, UK.; ^3^ The Roslin Institute and Royal (Dick) School of Veterinary Studies, University of Edinburgh, Easter Bush, Midlothian EH25 9RG, UK.; ^4^ Department of Biomedical Engineering, University of Utah, 36 S Wasatch Dr, Salt Lake City, UT 84112, USA.; ^5^ School of Biomedical Engineering & Imaging Sciences, King’s College London, London SE1 7EH, UK.; ^6^ Mauna Kea Technologies, 9, Rue d’Enghien, Paris 75010, France.

In the research article “Optical Detection of Distal Lung Enzyme Activity in Human Inflammatory Lung Disease” [1], the data availability statement was inadvertently omitted by the publisher. This has now been corrected in the PDF and HTML (full text).

## Data Availability

The authors confirm that the data supporting the findings of this study are available within the article and its Supplementary Materials.
