# Supplementary material for: Optical Detection of Distal Lung Enzyme Activity in Human Inflammatory Lung Disease
Source: BME Front. 2021 Apr 7:9834163. doi: 10.34133/2021/9834163 (PMC10530652; doi:10.34133/2021/9834163)
Supplement: Supplementary Materials — Supplementary Information containing supplementary Figures S1-6: chemistry materials and methods, patient data, toxicology studies, and stability studies. [file 9834163.f1.docx]

**Optical detection of distal lung enzyme activity in human inflammatory lung disease.**

**Supporting Material**

**Contents**

[1. Supplementary figures 3](#_Toc12280884)

[2. Chemistry Materials and Methods 8](#_Toc12280885)

[2.1. General 8](#_Toc12280886)

[2.2. Synthesis of FIB One 8](#_Toc12280887)

[2.3. MALDI analysis after tissue treatment 13](#_Toc12280888)

[3. Patient Data 14](#_Toc12280889)

[4. Toxicology studies 20](#_Toc12280890)

[4.1. FIB One 20](#_Toc12280891)

[4.2. AZD1236 22](#_Toc12280892)

[5. Stability studies 23](#_Toc12280893)

[6. References 25](#_Toc12280894)

# **Supplementary figures**


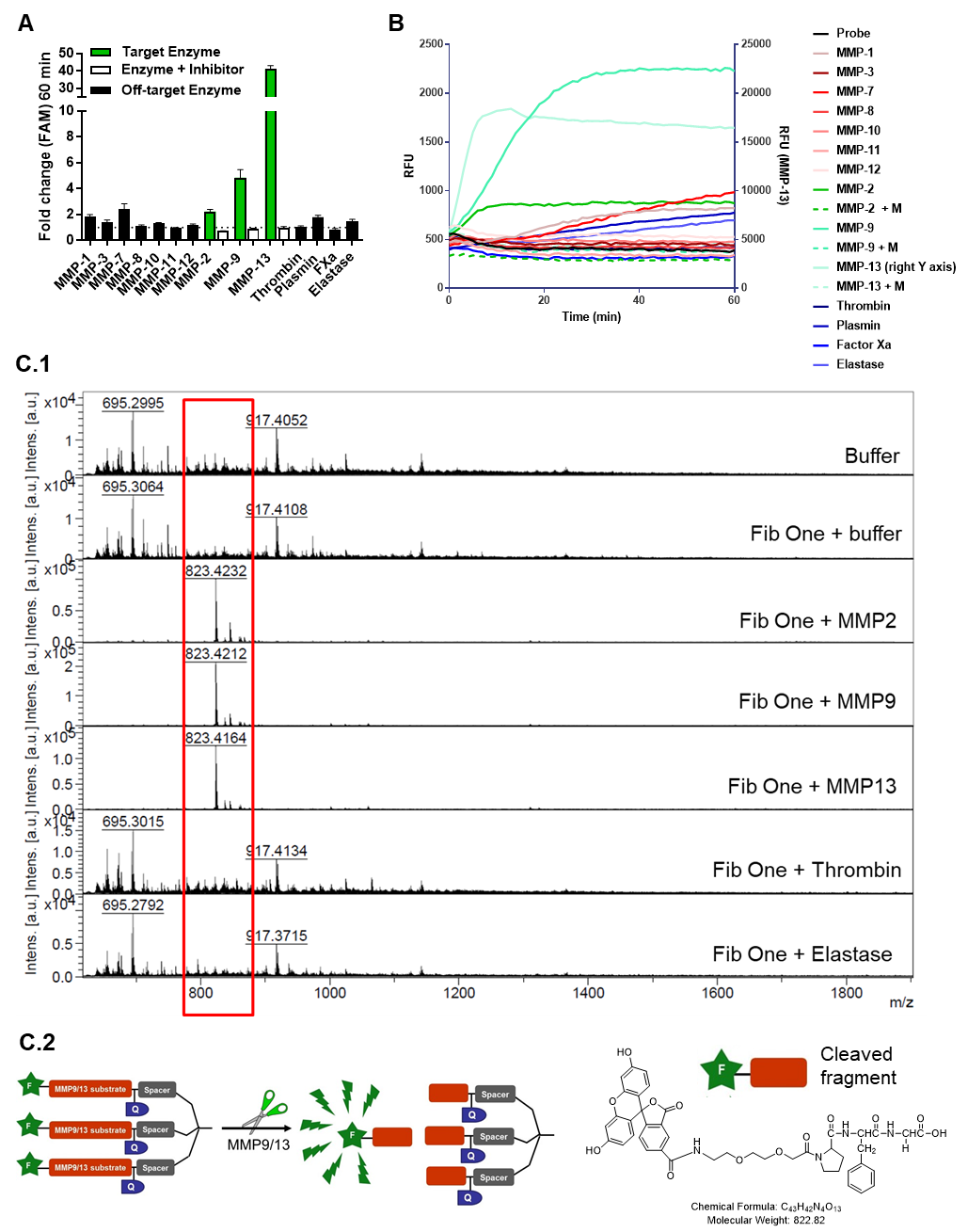


**Figure S1: *In vitro* testing of FIB One:** (A) Fold-change in fluorescent signal of FIB One (1 µM) following 60 min incubation with target (green), target plus inhibitor (marimastat, 20 µM, white) and off-target (black) enzymes, compared to enzyme-free control. [data shows mean and s.e.m. n = 3 (in duplicate), MMPs 30 nM, Thrombin 5 U/mL, Plasmin 30 nM, Factor Xa 500 nM, excitation/emission 485/528 nm]. (B) Representative time-course showing increase in fluorescence (RFU) of FIB One (1 µM) with target and off-target enzymes over 60 min. For clarity, the trace for MMP-13 is shown on the right-Y axis. (C.1) MALDI-TOF analysis of FIB One (1 µM) following 60 min incubation with target and off-target enzymes. (C.2) The peak at 823 Da indicated selective cleavage of FIB One as shown in the schematic diagram and structure of cleaved fragment.


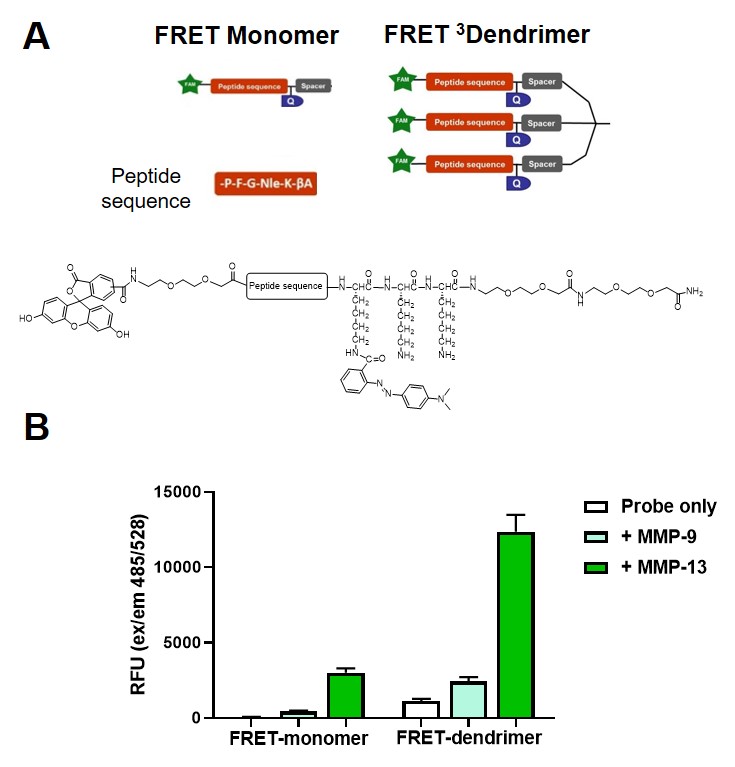


**Figure S2: Comparison of FRET-monomer vs FRET-dendrimer (FIB One).** (A) Full structure of FRET- monomer and schematic structures comparing FRET monomer and FRET ^3^Dendrimer (FIB One). (B) Fluorescent signal of FRET-monomer and FIB One (FRET-dendrimer) (1 µM) following 10 min incubation with MMP-9 and MMP-13. [data shows mean and s.e.m. n = 3 (in duplicate), MMPs 30 nM, excitation/emission 485/528 nm].


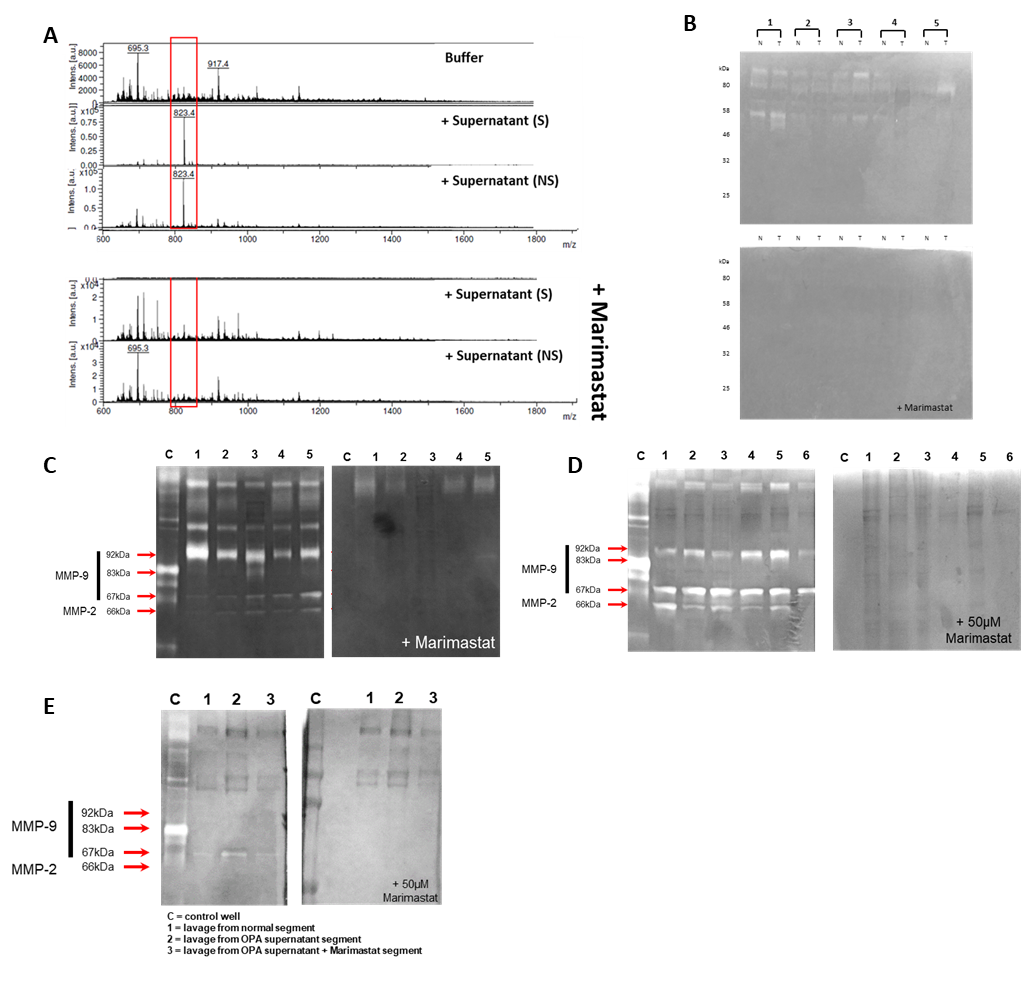


**Figure S3: *Ex vivo* validation of FIB One.** (A) MALDI-TOF analysis of 1 µM FIB One following 10 min incubation with stimulated and non-stimulated neutrophil supernatant. A peak at 823 Da indicated selective cleavage of FIB One. (B) Gelatin zymography of adjacent non-cancerous lung (N) and cancerous human lung (T) tissue samples 1-5. Identical gels were developed in the presence of marimastat (50 µM) to demonstrate MMP specificity of gelatin digestion. [Band sized observed: Pro-MMP-9 (92kDa), MMP-9 cleavage product or MMP-2 (67kDa) and MMP-2 activity (66kDa)]. (C) Gelatin zymography  of non-cancerous diseased human lung tissue samples 1-5 and control recombinant MMP-9 and pro-MMP-9, lane C. Identical gels were developed in the presence of marimastat (50 µM) to demonstrate MMP specificity of gelatin digestion. (D) Gelatin zymography of homogenised OPA tissue in lanes 1-6, representing six samples, demonstrating the presence of active MMPs. Identical gels were developed in the presence of Marimastat (50 µM) to demonstrate MMP specificity of gelatin digestion. (E) Gelatin zymography in the Ovine model, bronchoalveolar lavage was obtained from a segment without supernatant (lane 1) a segment instilled with OPA supernatant (lane 2) and a segment instilled with OPA supernatant and marimastat (lane 3). Gel on right in the presence of marimastat. [Band sized observed: Pro-MMP-9 (92kDa), MMP-9 cleavage product or MMP-2 (67kDa) and MMP-2 activity (66kDa)].


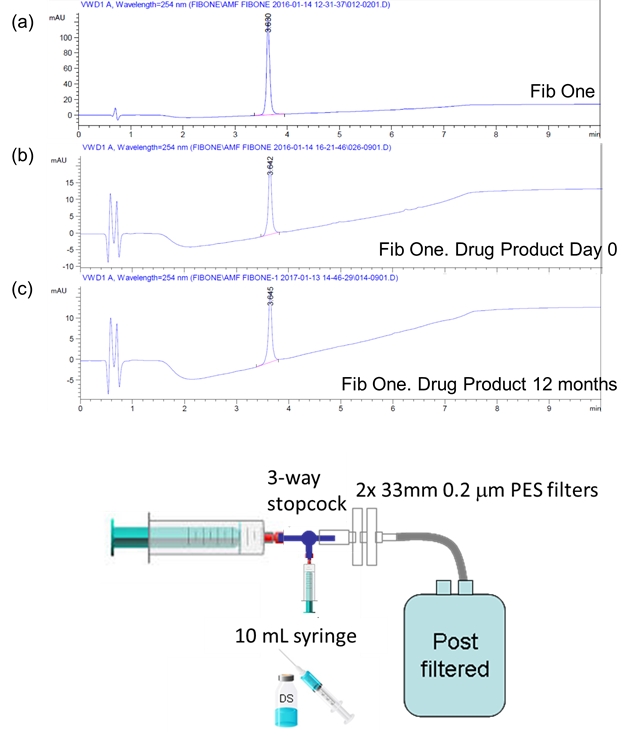


**Figure S4: Formulation and Stability:** FIB One was formulated into PBS at a concentration of less than 100 µg in 5 mL. FIB One Drug Substance (9 mg) was dissolved in 10 mL of H_2_O, double filtered (PES 0.2 mm) into a 600 mL transfer bag. The remaining PBS (490mL) was passed through the filters. The solution in the transfer bag was then divided into sterile vials, each containing approximately 5.2 mL of Drug Product.

1. HPLC chromatogram of FIB One (Drug Substance) with Detection at 254nm
2. HPLC chromatogram of FIB One (Drug Product) at Day 0
3. HPLC chromatogram of FIB One (Drug Product) after 12 months from formulation, where no signs of degradation were found. Storage at fridge temperature (~5^o^C).


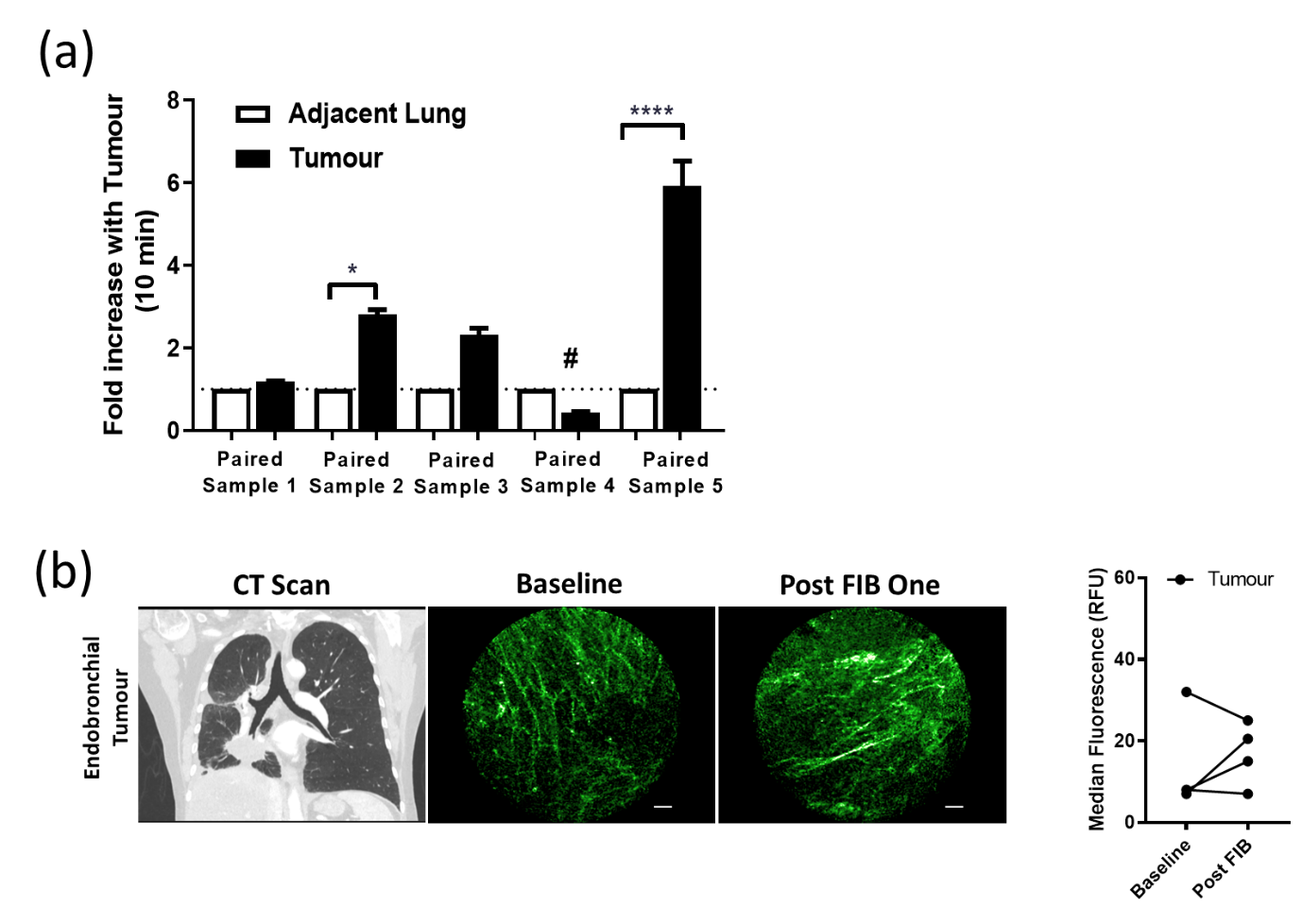


**Figure S5: Fib One imaging in human lung cancer ex vivo and in vivo.** (a) Fold-change in FIB One (1 µM) signal following 10 min incubation with ex vivo human NSCLC (tumour) and adjacent non-cancerous lung from surgical resections with excitation/emission 485/528 nm. Tumour fold-change for each patient was compared to adjacent lung tissue from the same patient. Data show mean (+/- SEM), n=3 performed in duplicate, analysis by one-way ANOVA, * p= 0.0472, **** p <0.0001. # tumour sample showed less MMP than adjacent lung sample by zymography (supplementary figure S3b). (b) Representative coronal CT image for an endobronchial tumour with baseline and post FIB One images on confocal endomicroscopy imaging. Quantification of in vivo human lung fluorescence measured using confocal endomicroscopy before and after bronchial delivery of the FIB One (n=4 for each group, p>0.05). Statistical analysis using a Mann Whitney test. Scale bar represents 50µm.


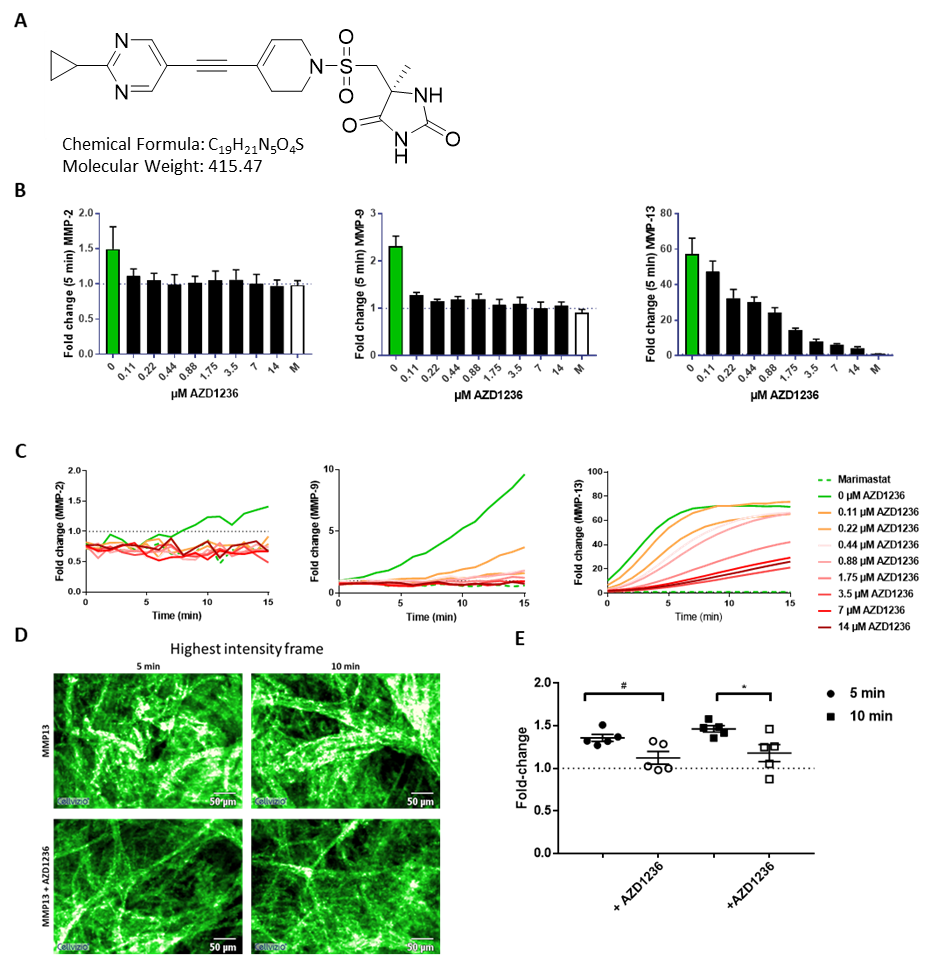


**Figure S6. *In vitro* and *ex vivo* analysis of MMP-inhibitor AZD1236 with FIB One.** (A) Chemical structure of AZD1236. (B) Fold-change in fluorescent signal of FIB One (1 µM) following 5 min incubation with MMP-2, 9 or 13 (30 nM) and increasing concentrations of AZD1236 (black bars, 0.11 – 14 µM) or marimastat (M, white bars, 20 µM), compared to enzyme-free controls. [data shows mean and s.e.m. n = 3 (in duplicate), excitation/emission 485/528 nm]. (C) Representative fold-change in fluorescent signal from FIB One (1 µM), measured every minute for 15 min following incubation with MMP-2, 9 or 13 (30 nM) and increasing concentrations of AZD1236 (0.11 – 14 µM) or marimastat (M, 20 µM), compared to enzyme-free controls. [data shows mean of one replica, completed in duplicate. Excitation/emission 485/528 nm]. (D) MMP13 (30nm) +/- AZD1236 (14 µM) or +/- marimastat (50 µM) was added to human lung tissue. FCFM imaging was performed (60s, 12 fps) prior to the addition of FIB One (1 µM). Imaging was repeated at 5 and 10 min post FIB One. The highest intensity from the video files are shown. (E) Data from each video file was quantified (703 frames collected per data set. Off-target frames were excluded). A mean intensity from each frame was collected. The median of these averages was calculated and the fold-change compared to time 0 was plotted. [Data shows mean and s.e.m, n=5. One-way ANOVA was performed. *P= 0.0227 ^#^P= 0.0737]

# **Chemistry Materials and Methods**

- 1. General

Commercially available reagents were used without further purification. Analytical reverse-phase high-performance liquid chromatography (RP–HPLC) was performed on an HP1100 system equipped with a reverse-phase column Kinetex XB-C18 (5 cm x 4.6 mm, 5 µm) with a flow rate of 1 mL/min and eluting with H_2_O/CH_3_CN/HCOOH (95/5/0.1) to H_2_O/CH_3_CN/HCOOH (5/95/0.1), over 6 min, holding at 95% ACN for 3 min, with detection at 254 nm. Preparative RP–HPLC was performed on an HP1100 system equipped with a Kinetex XB-C18, AXIA Packed reverse-phase column (150 x 21.2 mm, 5 μm, 100A) with a flow rate 10 mL/min and eluting with 0.1% HCOOH in H_2_O (A) and 0.1% HCOOH in CH_3_CN (B), with a gradient of 5 to 95% B over 18 min and additional isocratic period of 1 min. Electrospray ionization mass spectrometry (ESI–MS) analyses were carried out on an Agilent Technologies LC/MSD Series 1100 quadrupole mass spectrometer (QMS) in an ESI mode. MALDI spectra were acquired on a Bruker Ultraflextreme MALDI TOF/TOF, with a matrix solution of sinapic acid (10 mg/mL) in H_2_O/CH_3_CN/TFA (50/50/0.1).

2.2. Synthesis of FIB One

Scheme S1. Synthesis of FIB One

**2.2.1. H_2_N-Rink amide ChemMatrix resin (1):**

Peptide synthesis of branched compound was performed on Aminomethyl-ChemMatrix resin using 4-[(2,4-Dimethoxyphenyl)-(Fmoc-amino)methyl]phenoxyacetic acid (Rink amide linker) by the following procedure: Fmoc-Rink-amide (0.54g, 1.0 eq) was dissolved in DMF (10 mL) and Oxyma (0.14g, 1.0 eq.) was added and the mixture was stirred for 10 min. Diisopropylcarbodiimide (DIC, 155 µL, 1.0 eq.) was then added and the solution stirred for 1 min before adding it to Aminomethyl-ChemMatrix resin (1.0 g, 1.0 mmol/g). The resulting mixture was stirred at 50⁰C for 45 min and washed with DMF (3x10 mL), DCM (3x10 mL) and MeOH (3x10 mL). Finally the resin was treated with Ac_2_O:Py:DMF (2:3:15) for 30 min at rt in order to cap any remaining free amino group and it was washed again with DMF (3x10 mL), DCM (3x10 mL) and MeOH (3x10 mL). Resin loading[1] was calculated after that as ~0.58 mmol/g. The coupling reaction was monitored by ninhydrin test.[2] For the Fmoc deprotection, the resin (pre-swollen in DCM) was treated with 20% piperidine in DMF (5mL) and the reaction mixture was shaken for 10 min. The solution was drained and the resin was washed with DMF (3×10mL), DCM (3×10mL) and MeOH (3×10mL). This procedure was repeated twice. The deprotection reaction was monitored by the ninhydrin test.

**2.2.2. Isocyanate coupling to give (3):**

To resin (**1**) (0.30mmol), pre-swollen in DCM (10mL), was added a solution of isocyanate (**2**) [3] (920mg, 0.93mmol), DIPEA (0.2mL, 0.93mmol) and DMAP (22mg, 0.17mmol) in a mixture of DCM/DMF (1:1, 5mL) and the mixture was shaken overnight. The solution was drained and the resin was washed with DMF (3×20mL), DCM (3×20mL) and MeOH (3×20mL) and ether (3×20mL). (3×20mL). The coupling reaction was monitored by the ninhydrin test as described above.

**2.2.3.** **Synthetic steps on resin:**

**Dde deprotection of (VII):** To the resin pre-swollen in DCM was added 2% hydrazine in DMF and stirred at rt (5x10 min). The solution was drained and the resin washed with DMF (3x10 mL), DCM (3x10 mL) and MeOH (3x10 mL).

**General procedure for the Fmoc deprotection:** To the resin (pre-swollen in DCM) was added 20% piperidine in DMF (5mL) and the reaction mixture was shaken for 10 min. The solution was drained and the resin was washed with DMF (3×10mL), DCM (3×10mL) and MeOH (3×10mL). This procedure was repeated twice. The coupling reaction was monitored by the ninhydrin test.

**Pegylation ({2-[2-(Fmoc-amino)ethoxy]ethoxy}acetic acid or Fmoc-PEG_2_-OH):** A solution of Fmoc-PEG_2_-OH (3.0 eq per amine, 0.1M) and Oxyma (3.0 eq, 0.1M) in DMF was stirred for 10 min. DIC (3.0 eq, 0.1M) was added and stirred for 1 min. The pre-activated mixture was then added to the resin pre-swollen in DCM and the reaction heated at 50⁰C for 30 min. The solution was drained and washed with DMF (3x10 mL), DCM (3x10 mL) and MeOH (3x10 mL).

**Peptide Synthesis:** Peptide Sequence: -P-F-G-Nle-K-βA-

A solution of the appropriate Fmoc-amino acid (3.0mmol, 10 eq) [Fmoc-Lys(MethylRed)-OH, Fmoc-β−Ala-OH, Fmoc-Lys(Boc)-OH, Fmoc-Nle-OH, Fmoc-Leu-OH, Fmoc-Gly-OH, Fmoc-Phe-OH, Fmoc-Pro-OH] and Oxyma (3.0mmol, 10eq) was added and the mixture was stirred for 5-10 min. DIC (3.0mmol, 10 eq) was then added and the resulting mixture was stirred for a further 2 min. The solution was added to pre-swollen resin in DCM and the reaction mixture was mixed for 0.5h at 60°C. The solution was drained and the resin washed DMF (3×20mL), DCM (3×20mL) and MeOH (3×20mL). The coupling reactions were monitored by ninhydrin test as discussed above.

**5-Carboxyfluorescein (FAM) labelling:** A solution of FAM (3-10 eq) and oxyma (3-10 eq) in DMF (0.1 M) was stirred for 10-15 min. DIC (3-10 eq) was then added and the resulting solution was stirred for further 1-2 min. This solution was added to the appropriate resin (1 eq), pre-swollen in DCM, and the reaction mixture was stirred at 50°C for 30 min. The solution was drained and the resin washed with DMF (x3), DCM (x3) and MeOH **(**x3). The coupling reactions were monitored by ninhydrin test. Before cleavage, the resin was washed with 20% piperidine to remove any fluorescein phenol esters.

**TFA cleavage and purification:** The resin (600 mg), pre-swollen in DCM, was treated with a cleavage cocktail of TFA/TIS/H_2_O (95/2.5/2.5, 10mL) for 3h. The solution was drained and the resin was washed with the cleavage cocktail and added to ice-cold ether. The precipitated solid was collected by centrifugation and the solvent removed by decantation and the precipitate was washed with cold ether (3x50mL). The precipitate (372 mg) was then purified by preparative reverse phase HPLC and the desired fractions were pooled and lyophilized to afford product **FIB One** (101 mg, >95% purity) that was characterized by MALDI-TOF MS and analytical HPLC:

***FIB One***: HPLC t*_R_* = 3.59 min, MALDI calc. for C_324_H_453_N_62_O_78_ [M+H]^+^: 6465.3578; found: 6465.2218.

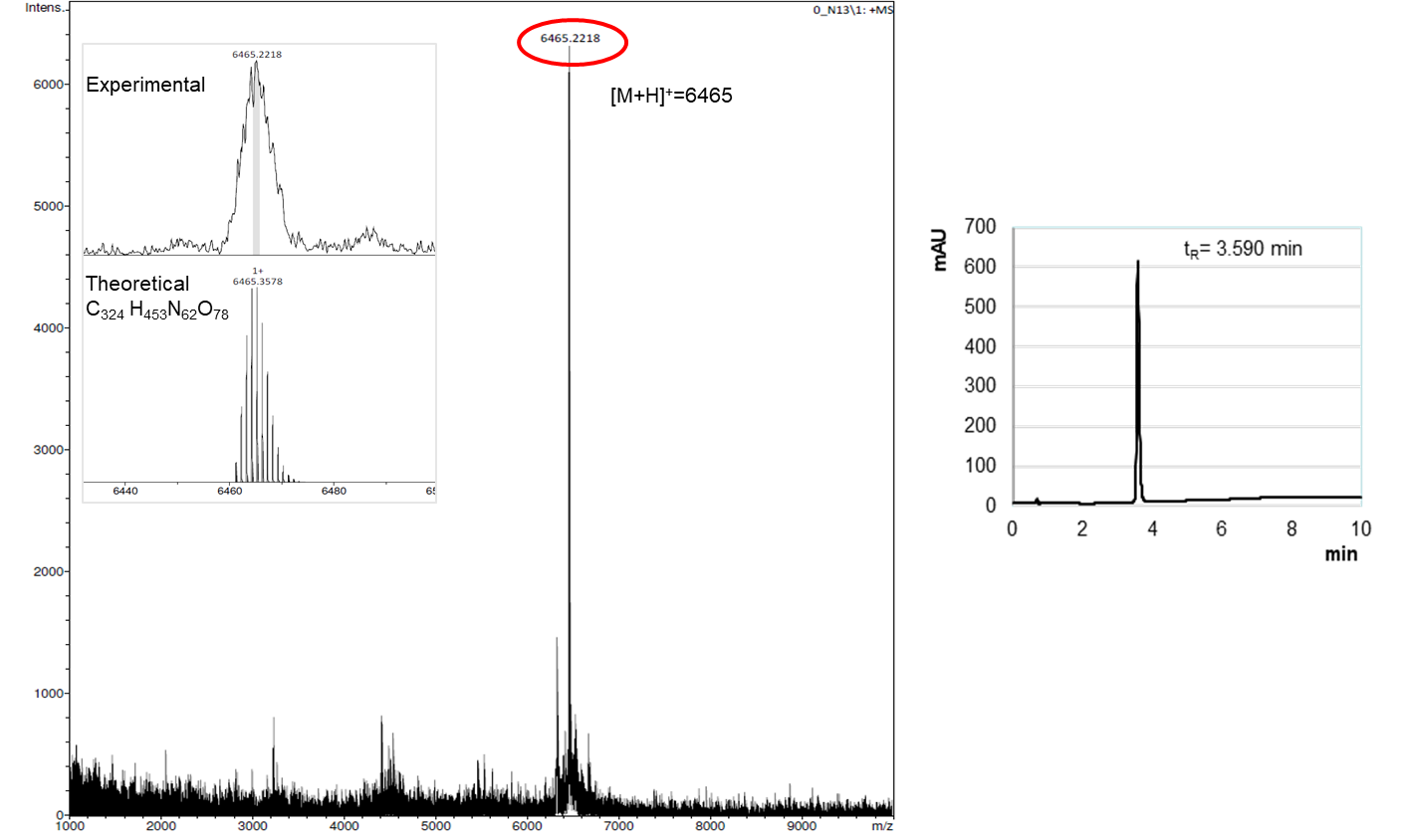


Figure S6. Structure, MALDI-TOF MS and RP-HPLC characterization of FIB One.

2.3. MALDI analysis after neutrophil treatment

Probe (1µM) was added to neutrophil supernatant and incubated for 10 minutes as indicated in Fig S3A. A ZipTip (C-18, 0.2 µL) was washed with 5µL Acetonitrile (with 0.1% TFA as an additive) followed by 20 µL of H_2_O. The ZipTip was loaded with the sample (10 µL), washed with H_2_O (2x 10 µL) and eluted into 5 µL of 80% aq. Acetonitrile (with 0.1% TFA as an additive). The sample was analysed by MALDI TOF/TOF, with a matrix solution of sinapic acid (10 mg/mL) in H_2_O/Acetonitrile/TFA (50/50/0.1).

# **Patient Data**

|  | **Baseline** | **Post Procedure** | **p value** |
| --- | --- | --- | --- |
| **Hb (g/dl)** | 148.6 (11.3) | 143.3 (12.3) | 0.0002 |
| **WCC (x10^9^/L)** | 7.6 (1.1) | 10.9 (2.9) | <0.0001 |
| **Platelets (x10^9^/L)** | 221.5 (53.2) | 221.2 (52.6) | 0.96 |
| **Sodium (mmol/L)** | 138.9 (2.7) | 137.7 (3.2) | 0.01 |
| **Potassium (mmol/L)** | 4.3 (0.4) | 4.2 (0.4) | 0.29 |
| **Urea (mmol/L)** | 4.6 (1.3) | 4.7 (1.1) | 0.34 |
| **Creatinine (umol/L)** | 77.5 (11.5) | 77.3 (12.1) | 0.85 |
| **Bilirubin (umol/L)** | 12.5 (5.3) | 12.4 (4.6) | 0.83 |
| **ALT (IU/L)** | 25.2 (18.2) | 23.8 (15.2) | 0.24 |
| **CRP (mg/L)** | 5.4 (8.1) | 5.2 (8.1) | 0.10 |

Table S1: Laboratory results. Displayed are the mean results (sd) from blood samples drawn pre procedure and 4-6 hours post FIB One delivery. Whilst there was a statistically significant change in haemoglobin, white cell count and sodium, there was no clinically significant alterations. Statistical analysis using paired t-test. (Hb= haemoglobin; WCC= white cell count; ALT- alanine transaminase; CRP= C Reactive Protein).

|  | Baseline | Hour 1 | Hour 2 | Hour 3 | Hour 4 |
| --- | --- | --- | --- | --- | --- |
| Temperature (C) | 36.6 (0.5) | 36.4 (0.4) | 36.4 (0.6) | 36.5 (0.4) | 36.8 (0.4) |
| Respiratory Rate (bpm) | 16 (1) | 16 (2) | 17 (1) | 16 (1) | 16 (1) |
| Systolic BP (mmHg) | 142 (22) | 124 (20) | 124 (18) | 122 (32) | 125 (17) |
| Oxygen Saturations (%) | 96 (1) | 97 (2) | 97 (2) | 96 (2) | 96 (2) |
| Heart Rate (bpm) | 76 (13) | 69 (13) | 67 (18) | 71 (12) | 77 (14) |

Table S2: Clinical observations. Displayed are the mean values at each timepoint (s.d.). Whilst there are minor fluctuations in values, between baseline and point of discharge from study (hour 4) there is no clinically significant variation.

| **Patient** | **Sex** | **Age** | **Diagnosis** | **Midazolam (mg)** | Alfentanyl (mc**g)** | **Fentanyl (**mc**g)** | **Adverse Events** |
| --- | --- | --- | --- | --- | --- | --- | --- |
| **DO01** | M | 73 | NSCLC | 3 | 300 |  | Haemoptysis |
| **DO02** | F | 79 | SCLC | 1 | 300 |  | Nil |
| **DO03** | M | 65 | IPF | 2.5 | 700 |  | Nil |
| **DO04** | F | 73 | NSCLC | 4 | 500 |  | Cough |
| **DO05** | M | 54 | SCLC | 3.5 | 300 |  | Fever |
| **DO06** | M | 74 | IPF | 2 |  | 75 | Nil |
| **DO07** | M | 58 | Chronic HP | 3 |  | 50 | Nil |
| **DO08** | M | 75 | RA-ILD | 3 | 300 |  | Nil |
| **Mean** |  | 69 |  | 2.75 | 400 | 62.5 |  |

Table S3: Patients demographics and AEs Part A. Demographics of patients from Part A of the FIB One study. (NSCLC= Non Small Cell Lung Cancer; SCLC= Small Cell Lung Cancer; IPF= Idiopathic Pulmonary Fibrosis; Chronic HP= Chronic Hypersensitivity Pneumonitis; RA-ILD= Rheumatoid Arthritis related Interstitial Lung Disease).

| **Patient** | **Condition** | **Total No. of Frames** | **No. of Acceptable Frames** |
| --- | --- | --- | --- |
| **DO01** | Tumour Bronchus Pre FIB | 1087 | 254 (23%) |
| **DO02** | Tumour Bronchus Pre FIB | 1403 | 530 (38%) |
| **DO03** | Fibrotic Alveoli Pre FIB | 1434 | 439 (31%) |
| **DO04** | Control Alveoli Pre FIB | 743 | 184 (25%) |
|  | Tumour Bronchus Pre FIB | 787 | 303 (39%) |
| **DO05** | Control Alveoli Pre FIB | 782 | 425 (54%) |
|  | Tumour Bronchus Pre FIB | 2325 | 1561 (67%) |
| **DO06** | Fibrotic Alveoli Pre FIB | 1230 | 708 (58%) |
| **DO07** | Fibrotic Alveoli Pre FIB | 4514 | 4052 (90%) |
| **DO08** | Fibrotic Alveoli Pre FIB | 6949 | 5828 (84%) |

Table S4: Quality Assessment of videos: Part A. Imaging quality assessment on a frame by frame basis. All videos were reviewed by two assessors and frames removed if they showed lack of structure, excessive movement or non relevant structure (e.g. bronchial imaging when assessing alveolar fluorescence). All tumor imaging was performed by applying the fibre tip to bronchial tumour under direct vision. All alveolar imaging was performed as a blind transbronchial pass.

| **Patient** | **Sex** | **Age** | **Diagnosis** | **Midazolam (mg)** | **Alfentanyl (mcg)** | **Adverse Events** |
| --- | --- | --- | --- | --- | --- | --- |
| **DO09** | F | 75 | Chronic HP | 3 | 300 | Cough |
| **DO10** | M | 69 | SScrILD | 4 | 300 | Nil |
| **DO11** | M | 79 | IPF | 3 | 200 | Nil |
| **DO12** | M | 73 | IPF | 5 | 300 | Cough  Fever |
| **DO13** | M | 61 | NSCLC | 3 | 500 | Haemoptysis |
| **DO14** | M | 57 | Chronic HP | 5 | 600 | Nil |
| **DO15** | M | 76 | Idiopathic NSIP | 3 | 400 | Nil |
| **DO16** | M | 61 | IPF | 4 | 400 | Nil |
| **DO17** | F | 50 | CTrILD | na | na | Nil |
| **Mean** |  | 67 |  | 3.75 | 375 |  |

Table S5: Patient demographics and AEs part B. Three patients reported adverse events within the 24h period post procedure all of which are commonly associated with flexible bronchoscopy. Two patients complained of a minor cough which was spontaneously resolving, one patient described an episode of fever later in the evening post procedure (this patient had had a broncheoalveolar lavage) and one patient had minor haemoptysis in the context of endobronchial biopsy performed at the same procedure.

| **Patient** | **Segment** | **Usable?** | **Reason if not useable** | **% of segments usable** |
| --- | --- | --- | --- | --- |
| **DO09** | A | Yes |  | 75% |
|  | B | No | Poor Quality,  Movement |  |
|  | C | Yes |  |  |
|  | D | Yes |  |  |
| **DO10** | A | Yes |  | 60% |
|  | B | No | Movement due to coughing |  |
|  | C | No | Poor Quality |  |
|  | D | Yes |  |  |
|  | E | Yes |  |  |
| **DO11** | A | Yes |  | 100% |
|  | B | Yes |  |  |
|  | C | Yes |  |  |
|  | D | Yes |  |  |
| **DO12** | A | Yes |  | 66.6% |
|  | B | Yes |  |  |
|  | C | Yes |  |  |
|  | D | Yes |  |  |
|  | E | No | Not enough delay after AZ delivery |  |
|  | F | No | Excessive coughing-movement |  |
| **DO13** | A | No | Excessive movement- respiration | 0% |
|  | B | No | Excessive movement- respiration |  |
|  | C | No | Excessive movement- respiration |  |
| **DO14** | A | Yes |  | 100% |
|  | B | Yes |  |  |
|  | C | Yes |  |  |
|  | D | Yes |  |  |
| **DO15** | A | Yes |  | 100% |
|  | B | Yes |  |  |
|  | C | Yes |  |  |
|  | D | Yes |  |  |
| **DO16** | A | No | Movement after FIB delivery | 75% |
|  | B | Yes |  |  |
|  | C | Yes |  |  |
|  | D | Yes |  |  |
| **DO17** | A | Yes |  | 100% |
|  | B | Yes |  |  |

Table S6: Quality Assessment of videos Part B. Quality assessment of segmental imaging. All videos were assessed by two assessors to determine if they were of sufficient quality for analysis. If there was significant movement of field of view between videos in a segment, this segment was removed from analysis.


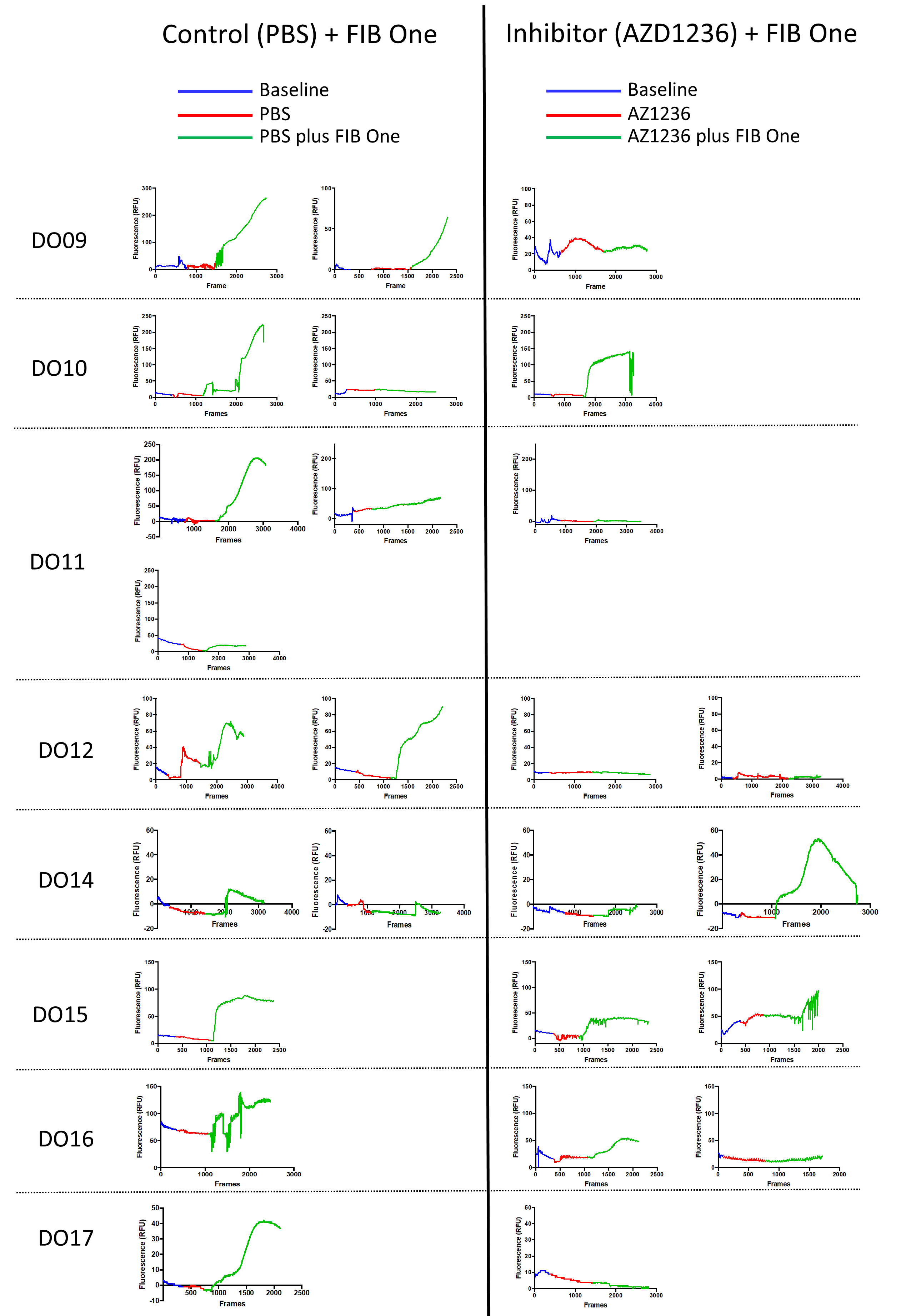


**Figure S7**. All fluorescence change in Part B. Mean fluorescence intensity over time during alveolar delivery of PBS, AZ and FIB One Real-time consecutive measurement of fluorescence in 26 pulmonary segments. (PBS= phosphate buffered saline; AZ= AZD1236)

1. Toxicology studies
   1. **FIB One**

Assessment of toxicity of FIB One was based on clinical signs, body weight, food consumption and clinical and anatomic pathology evaluations. Complete necropsies were performed on all animals with a recording of macroscopic abnormalities for all tissues. Organ weights and microscopic examinations were conducted as indicated.

There were no deaths, no adverse clinical signs and no effects of treatment with FIB One on body weight, food consumption or clinical or anatomic pathology.

In conclusion, a single intratracheal administration of FIB One to Crl:WI(Han) rats at 100 µg/animal (500 µg/mL) was well tolerated with no evidence of acute or delayed toxicity.

**
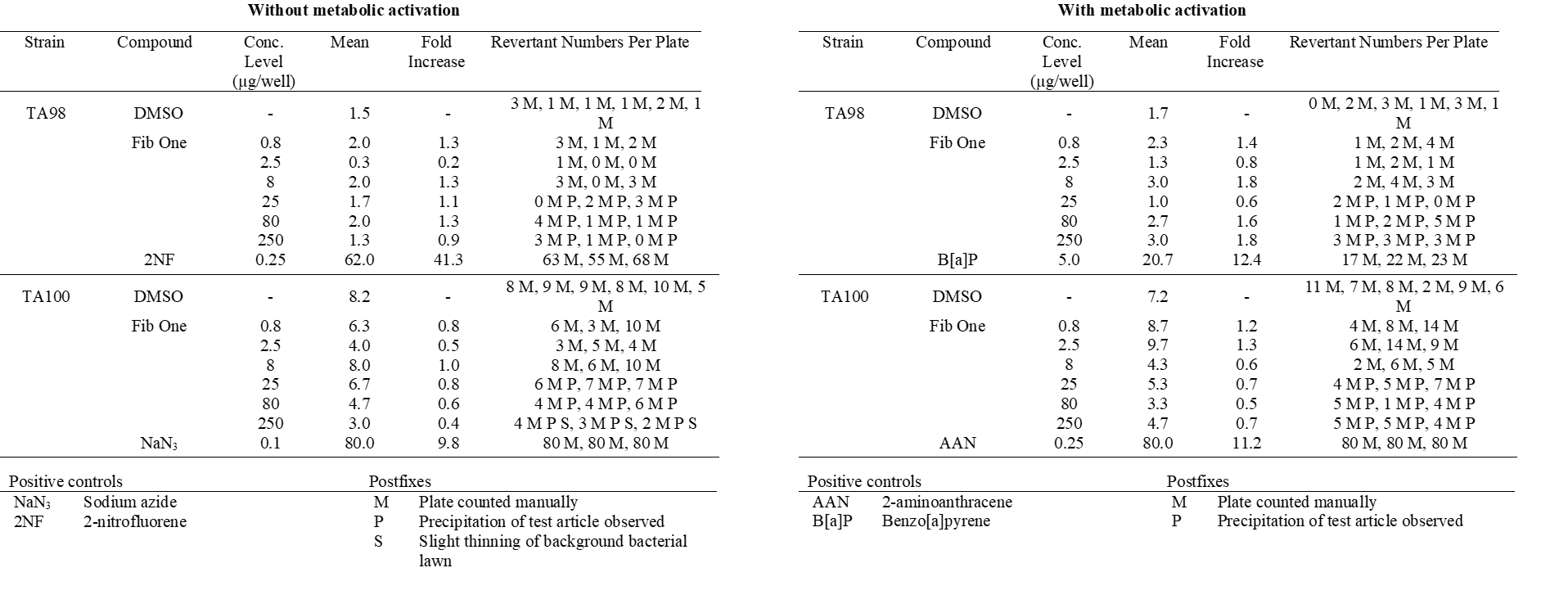
**

**
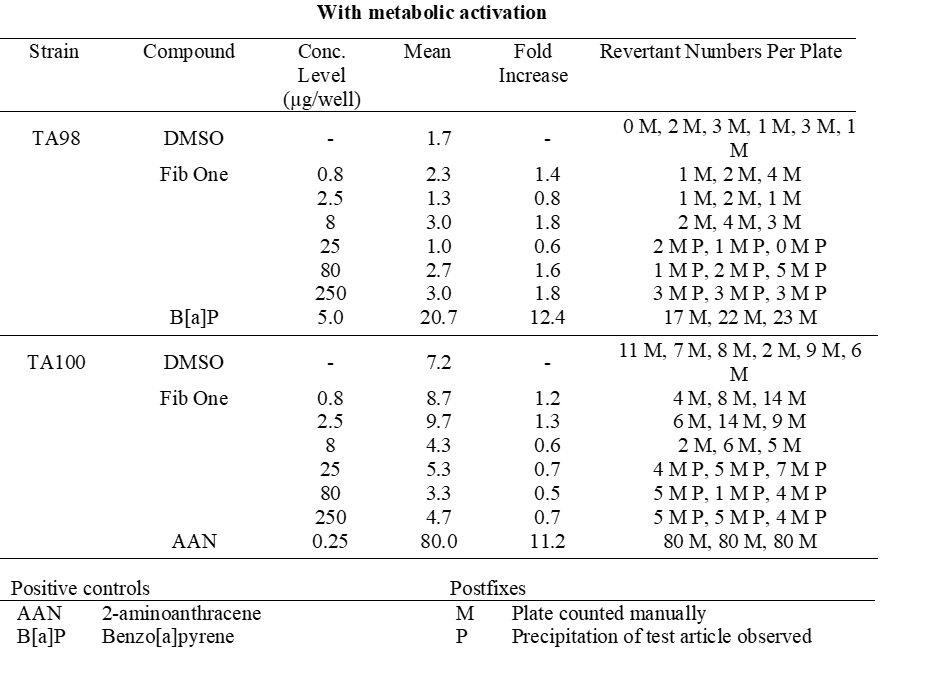
**

**Table S7: FIB One AMES test:** FIB One does not induce mutation in *Salmonella typhimurium* in a reduced test article AMES test. It was concluded that FIB One did not induce mutation in *Salmonella typhimurium* strains TA98 and TA100 when tested under the conditions of this study. These conditions included treatments at concentrations up to 250 μg/well (a 20-fold pro rata reduction from the standard regulatory Ames assay maximum concentration), in the absence and in the presence of a rat liver metabolic activation system (S-9).

- 1. AZD1236

Assessment of toxicity of AZD1236 was based on clinical signs, body weight, food consumption, clinical and anatomic pathology evaluations. Complete necropsies were performed on all animals with a recording of macroscopic abnormalities for all tissues. Organ weights and microscopic examinations were conducted as indicated.

There were no deaths, no adverse clinical signs and no effects of treatment with MRC AZD 1236 on body weight, food consumption or clinical or anatomic pathology.

Degeneration of the tracheal bifurcation in some animals, including controls, was attributed to minor tissue damage from the intratracheal dosing method and was only noticeable on Day 2, shortly after performing the procedure. This finding was not present at Day 15, suggesting complete resolution of any damage.

In conclusion, a single intratracheal administration of MRC AZD1236 to Crl:WI(Han) rats at 35 µg/animal (70 µg/mL) was well tolerated with no evidence of acute or delayed toxicity.

1. Stability studies

| **Conditions**      **Timepoint** | | **Fridge 2-8 °C** | | | |
| --- | --- | --- | --- | --- | --- |
|  |  | **RT / min** | **Conc. / μg per 5mL (∆%)** | **Total imp. RRT(%)** | **pH** |
| 0 | (14JAN2016) | RT: 3.636 min / pH: 7.5 | | | |
| 24h | (15JAN2016) | 3.659  3.654 | 81.63 (-9.3)  82.28 (-8.6) | ND/NQ  ND/NQ | 7.5  7.5 |
| 14d | (28JAN2016) | 3.666  3.667 | 76.85 (-14.6)  84.60 (-6.1) | ND/NQ  ND/NQ | 7.5  7.5 |
| 1m | (14FEB2016) | 3.651  3.651 | 80.16 (-10.9)  77.20 (-14.2) | ND/NQ  ND/NQ | 7.5  7.5 |
| 3m | (14APR2016) | 3.643  3.644 | 78.87 (-12.4)  72.63 (-19.3) | ND/NQ  ND/NQ | 7.5  7.5 |
| 6m | (07JUL2016) | 3.652  3.656 | 64.94 (-27.8)  72.74 (-19.2) | ND/NQ  ND/NQ | 7.5  7.5 |
| 9m | (14OCT2016) | 3.671  3.674 | 70.54 (-21.6)  66.27 (-26.4) | ND/NQ  ND/NQ | 7.5  7.5 |
| 10m | (16NOV2016) | 3.647  3.648 | 62.55 (-30.5)  67.69 (-24.8) | ND/NQ  ND/NQ | 7.5  7.5 |
| 12m | (14JAN2017) | 3.651  3.647 | 60.40 (-32.9)  57.67 (-35.9) | ND/NQ  ND/NQ | 7.5  7.5 |
| 21m | (18OCT2017) | 3.651  3.647 | 65.38 (-27.3)  59.62 (-33.8) | ND/NQ  ND/NQ | 7.5  7.5 |
| 24m | (14JAN2018) | 3.618  3.618 | 58.4 (-35.1)  58.4 (-35.1) | ND/NQ  ND/NQ | 7.5  7.5 |

**Table S8:** **FIB One remains biologically functional in aqueous formulation for 24 months.** Summary table of stability as assessed by HPLC over 24 months stored under GMP conditions at 4-8°C. Data shown in blue represents inverted position vials. Δ% is relative to starting concentration 90μg/5mL at time zero. Acceptance criteria: Retention time (RT) for FIB One is 3.65 min (Range 3.40-3.80 min). Total impurities – report as RRT (%) for each impurity. Biological functionality LOD = >20μg/5mL. pH 7.5 (7.0-8.0).


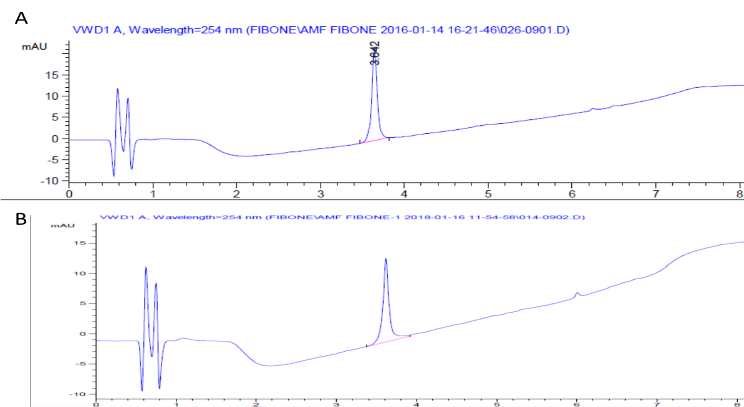


**Figure S8:** HPLC traces of FIB One at time zero (A) and after 24 months (B) showing no degradation in aqueous formulation of the drug product when stored at 2-8°C

| **Conditions**      **Timepoint** | | **Room temperature** | | | |
| --- | --- | --- | --- | --- | --- |
|  |  | **RT / min** | **Conc. / μg per 5mL (∆%)** | **Total imp. RRT(%)** | **pH** |
| 0 | (05APR2016) | RT: 3.980 min  pH: 7.5 | | | |
| 24h | (06APR2016) | 3.979  3.980 | 74.3 (+6.0)  74.5 (+6.0) | ND/NQ  ND/NQ | 7.5  7.5 |
| 15d | (20APR2016) | 3.983  3.981 | 70.2 (+0.14)  70.3 (+0.21) | ND/NQ  ND/NQ | 7.5  7.5 |
| 1m | (05MAY2016) | 3.981  3.980 | 77.0 (+10.0)  76.8 (+9.70) | ND/NQ  ND/NQ | 7.5  7.5 |
| 3m | (05JUL2016) | 3.982   3.981 | 69.4 (-0.90)   69.4 (-0.90) | ND/NQ   ND/NQ | 7.5   7.5 |
| 6m | (05OCT2016) | 3.975  3.984 | 70.8 (+1.00)  70.6 (+0.80) | ND/NQ  ND/NQ | 7.5  7.5 |
| 10.5m | (17FEB2017) | 3.972  3.972 | 71.1 (+1.50)  70.7 (+1.00) | ND/NQ  ND/NQ | 7.5  7.5 |
| 12m | (05APR2017) | 3.977  3.977 | 70.0 (+0.00)  69.2 (-1.15) | ND/NQ  ND/NQ | 7.5  7.5 |
| 18m | (05OCT2017) | 3.974  3.975 | 70.4 (+0.6)  70.2 (+0.3) | ND/NQ  ND/NQ | 7.5  7.5 |
| 24m | (05APR2018) | 3.968  3.973 | 72.96 (+4.2)  72.52 (+3.6) | ND/NQ  ND/NQ | 7.5  7.5 |

**Table S9:** AZD1236 remains biologically functional in aqueous formulation for 24 months. Summary table of stability as assessed by HPLC over 24 months stored under GMP conditions at RT. Data shown in blue represents inverted position vials. ∆% is relative to starting concentration of 70μg/5mL at time zero. Acceptance criteria: Retention time (RT) for AZD1236 is 3.97 min (Range 3.40-4.50 min). Total impurities – report as RRT (%) for each impurity. Biological functionality LOD = >21μg/5mL. pH 7.5 (7.0-8.0).


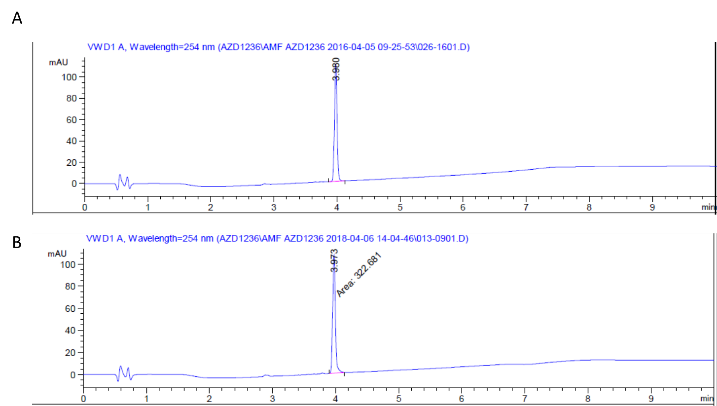


**Figure S9:** HPLC traces of AZD1236 at time zero (A) and after 24 months (B) showing no degradation in aqueous formulation of the drug product when stored at ambient temperature.

1. References

1. Gude, M., J. Ryf, and P.D. White, An accurate method for the quantitation of Fmoc-derivatized solid phase supports. *Lett. Pept. Sci.*, **2002**. 9(4-5) 203-206.

2. Kaiser, E., R. Colescott, C. Bossinger, and P. Cook, Color test for detection of free terminal amino groups in the solid-phase synthesis of peptides. *Analytical Biochemistry,* **1970**. 34(2) 595.

3. Avlonitis, N., M. Debunne, T. Aslam, N. McDonald, C. Haslett, K. Dhaliwal, and M. Bradley, Highly specific, multi-branched fluorescent reporters for analysis of human neutrophil elastase. *Org. Biomol. Chem.*, **2013**. 11(26) 4414-4418.
